# CD63^+^ and MHC Class I^+^ Subsets of Extracellular Vesicles Produced by Wild-Type and CD47-Deficient Jurkat T Cells Have Divergent Functional Effects on Endothelial Cell Gene Expression

**DOI:** 10.3390/biomedicines9111705

**Published:** 2021-11-17

**Authors:** Sukhbir Kaur, Abdel G. Elkahloun, Jennifer D. Petersen, Anush Arakelyan, Ferenc Livak, Satya P. Singh, Leonid Margolis, Joshua Zimmerberg, David D. Roberts

**Affiliations:** 1 Laboratory of Pathology, Center for Cancer Research, National Cancer Institute, National Institutes of Health, Bethesda, MD 20892, USA; 2 Cancer Genetics Branch, National Human Genome Research Institute, National Institutes of Health, Bethesda, MD 20892, USA; abdel@nhgri.nih.gov; 3 Section on Integrative Biophysics, Division of Basic and Translational Biophysics, Eunice Kennedy Shriver National Institute of Child Health and Human Development, National Institutes of Health, Bethesda, MD 20892, USA; jennifer.petersen@nih.gov (J.D.P.); zimmerbj@mail.nih.gov (J.Z.); 4 Section on Intercellular Interactions, Eunice Kennedy Shriver National Institute of Child Health and Human Development, National Institutes of Health, Bethesda, MD 20892, USA; anush.arakelyan@gmail.com (A.A.); margolil@mail.nih.gov (L.M.); 5 Flow Cytometry Core, Laboratory of Genome Integrity, Center for Cancer Research, National Cancer Institute, National Institutes of Health, Bethesda, MD 20892, USA; ferenc.livak@nih.gov; 6 Inflammation Biology Section, Laboratory of Molecular Immunology, National Institute of Allergy and Infectious Diseases, National Institutes of Health, Bethesda, MD 20892, USA; spsingh@niaid.nih.gov

**Keywords:** extracellular vesicles, non-coding RNAs, cell-cell communication

## Abstract

T cells and endothelial cells engage in bidirectional communication that regulates angiogenesis and T cell transmigration. Extracellular vesicles (EVs) mediate intercellular communication by the transfer of bioactive molecules including RNAs. EVs produced by a given cell type are heterogeneous in their RNA content, but it is unclear how specific EV surface markers relate to their functional effects on target cells. Our previous work established that Jurkat T cell EVs bearing CD63, MHC-I, or CD47 surface markers contain distinct noncoding RNA populations. The present study reveals that CD63^+^ and MHC-I^+^ EVs from CD47-deficient Jurkat T cells are enriched in small non-coding RNAs relative to EVs from wild-type Jurkat T cells. CD47-deficient Jurkat T cells secrete more CD63^+^ and MHC-I^+^ EVs, but MHC-I^+^ EVs are selectively taken up more by human umbilical vein endothelial cells. Transcriptomics analysis of endothelial cells treated with CD63^+^ or MHC-I^+^ EVs showed surface marker- and CD47-dependent changes in gene expression in the target cells. Gene set enrichment analysis identified CD47-dependent, and surface marker-dependent effects of T cell EVs on VEGF and inflammatory signaling, cell cycle, and lipid and cholesterol metabolism. Thus, subsets of T cell EVs differentially regulate endothelial cell metabolism and inflammatory and angiogenic responses.

## 1. Introduction

Extracellular vesicles play important roles in cell-cell communication, mediated in part by the transfer of mRNAs and miRNAs via several uptake mechanisms [1,2,3] that alter the fate of acceptor cells [4,5,6]. EVs are heterogeneous and bear various surface markers, including CD63, major histocompatibility complex class-I (MHC-I), and CD47 [7,8,9,10]. We showed previously that exposing human umbilical vein endothelial cells (HUVEC) to total EVs produced by wild-type (WT) Jurkat T cells or mutant Jurkat T cells lacking CD47 (JinB8) alter HUVEC gene expression and differentially modulate VEGF-induced cell proliferation, tube formation, and VEGFR2 phosphorylation [11]. In contrast, treatment of HUVEC with EVs released by breast carcinoma cells regulate endothelial–mesenchymal transition by altering expression of Zeb1 and integrins in a CD47-dependent manner [12]. Noncoding RNAs identified in the breast carcinoma EVs were consistent with the endothelial cell responses being mediated by the transfer of these EV RNAs into HUVEC.

In addition to divergent effects of EVs on endothelial cells based on their cell of origin, individual cells may produce multiple subsets of EVs that have divergent contents and effects on target cells. We found that subsets of WT Jurkat T cell EVs purified based on the surface markers CD47, CD63, and MHC-I contain distinct small RNAs [13]. Their divergent RNA compositions suggest distinct functions in mediating T cell communication with target cells. In this study, we compared the effects on HUVEC gene expression of CD63^+^ and MHC-I^+^ EV subsets derived from WT Jurkat and JinB8 T cells and identify several functional gene families in HUVEC that are differentially regulated by these EV populations. Furthermore, differences in miRNAs in the EVs were consistent with their effects on HUVEC gene expression that regulate angiogenic, metabolic, and inflammatory responses.

## 2. Materials and Methods

### 2.1. Materials

WT Jurkat and JinB8 T cells [14] were cultured using RPM1 1640 medium supplemented with 10% FBS (Gemini Bio, West Sacramento, CA, USA), glutamine and penicillin/streptomycin (Thermo Fischer Scientific, Waltham, MA, USA) as described previously [15]. Purified anti-human HLA-C Antibody, antibody, anti-human CD63 antibody, Biotin anti-human CD63 antibody, biotin anti-human HLA-A, B, C antibody, MHC-I-APC/640, CD63-PE and CD47-FITC antibodies were purchased from BioLegend, San Diego, CA, USA.

### 2.2. EV Extraction

WT Jurkat and JinB8 T cells were cultured in serum-free HITES medium as described previously [15]. In brief, ~1 × 10^6^ WT Jurkat and JinB8 T cells were cultured overnight with 50 mL of HITES media using 175 cm^2^ flasks in triplicate for each cell type. Cells were centrifuged at 300× *g* for 5 min, and the conditioned media were transferred into new tubes. The cell free conditioned media was further centrifuged at 2000× *g* for 10 min to remove cell debris. The cell conditioned media of each flask were concentrated using 15 mL amplicon with 10,000 MW cutoff to a volume of 1 mL. The concentrated media was pooled together and centrifuged at 16,000× *g* for 30 min, and EVs were purified using Exo-spin Exosome Purification with size exclusion chromatography (SEC) from Cell Guidance System according to the manufacturer’s instructions.

### 2.3. Immunocapture of EVs

For small RNA sequencing, the total EVs released from WT Jurkat and JinB8 T cells were incubated with 15 nm magnetic nanoparticles (MNPs) bearing CD63 or MHC-I antibodies. For microarray analysis and HUVEC uptake assays, CD63^+^ and MHC-I^+^ EVs released from WT Jurkat and JinB8 cells were captured using 50 nm Super Mag Streptavidin Beads (Ocean NanoTech LLC, San Diego, CA, USA) with biotinylated antibodies as described previously [13]. In brief, EVs from WT Jurkat and JinB8 T cells were extracted using Exo-spin E exosome Purifications as described above. The final volume of each EV extraction was 200 μL per replicate. The extracted EVs (100 μL) were incubated with MHC-I and CD63 antibodies at 4 °C for 1 h. Super Mag Streptavidin Beads (50 nm) from Ocean NanoTech LLC were washed 3 times with PBS using a magnetic rack on ice. Super Mag Streptavidin Beads require 10–15 min settling time on the magnetic rack, and pelleted beads were dissolved in 100 μL of PBS and added to EV–antibody complexes for 30 min on ice. Beads with bound MHC-I^+^ and CD63^+^ EVs were washed 3 times with 1 mL of PBS without disturbing the pelleted EV bead complex on a magnetic rack. The EV–bead complexes were dissolved into a final volume of 100 μL per replicate. A 20 μL volume of EV–bead complex was used from each replicate in a 12-well plate containing HUVEC with 500 μL of media for microarray, real-Time PCR, and cytokine analyses.

### 2.4. EV Size Characterization

Conditioned media from WT Jurkat and JinB8 T cells were prepared using HITES media as described in the EV extraction method. The size distribution of EVs was analyzed using the Nanoview platform from concentrated conditioned media derived from WT Jurkat and JinB8 T cells as described previously [16].

### 2.5. Small RNA Sequencing Analysis

EVs were extracted using Exo-spin Exosome Purification kit and immunocaptured as described above. Captured and uncaptured fractions of CD63^+^ and MHC-I^+^ EVs from WT Jurkat cells (GSE103493) were compared with Captured fraction of CD63+ and MHC-I^+^ EVs from JinB8 T cells using Small RNA Sequencing Analysis workflow as described [13].

### 2.6. Microarray Expression and Gene Set Enrichment Analysis (GSEA)

HUVEC (ATCC) were co-cultured with immunocaptured CD63^+^ and MHC-I^+^ EVs or control beads alone from WT Jurkat and JinB8 T cells for 3 days. The total RNA from HUVEC was extracted using miRNA easy kit equal amount of RNAs was subjected to global microarray analyses. Gene Set Enrichment Analysis (GSEA, gsea-msigdb.org) was performed as described previously [11]. GSEA used data sets for HUVEC treated with either WT Jurkat MHC-I^+^ versus JinB8 MHC-I^+^ EVs or WT Jurkat CD63^+^ versus JinB8 CD63^+^. GSEA analysis reports are included in the supplemental HTML file.

### 2.7. EV Uptake

Freshly prepared EVs were enriched as described above and labeled with pHrodo™dye (Invitrogen) as previously shown [12]. The volume of the EV–bead complex for EV uptake assay, in triplicate, was adjusted based on the final volume in 96-well plates and blank (no beads) used as UT control. pHrodo™ dye was used to observe uptake of EVs by HUVEC measured using cell image cytometry (Celigo, Lawrence, MA, USA).

### 2.8. Incucyte EV Uptake Assay

Fresh CD63^+^ and MHC-I^+^ EVs were extracted as described above, labeled using pHrodo™dye, and co-cultured with HUVEC in 96-well plates (Corning, Tewksbury, MA, USA) with 6 replicates for each treatment for live cell analysis using the IncuCyte S3 (Essen BioScience, Ann Arbor, MI, USA). EV uptake was measured based on percent of red intensity and significance assessed via *t* test.

### 2.9. Cytokine Analysis

HUVEC were plated overnight in 12-well plates. CD63^+^ and MHC-I^+^ EVs isolated, as described in Section 2.3, were added to the HUVEC and incubated for 24–48 h. The HUVEC conditioned media was harvested and analyzed for TNFα (*n* = 2). We evaluated TNFα using an in-house multiplexed bead-based assay as previously described [17]. Briefly, antibody pairs and cytokine standards were purchased from R&D Systems. Luminex magnetic bead sets (Luminex, Riverside, CA, USA) were coupled to cytokine-specific capture antibody anti-TNFα (MAB610) according to the manufacturer’s recommendations. Conjugated beads were washed and kept at 4 °C until use. Biotinylated polyclonal antibodies were used at 100 ng/mL. All assay procedures were performed in assay buffer containing PBS supplemented with 1% normal mouse serum (GIBCO, Waltham, MA, USA), 1% normal goat serum (GIBCO), and 20 mM Tris-HCl (pH 7.4). The assays were run using 1200 beads per well in a total volume of 50 µL, in duplicate. Harvested conditioned media of cells was added to the wells containing cytokine capture beads and incubated for 2 h on a shaker. After, the plate was washed on a magnetic washer twice with 200 µL of assay buffer. The beads were then resuspended in 50 µL of assay buffer containing biotinylated polyclonal antibodies against TNFα (BAF210) and incubated for 1 h at room temperature. Plates were washed again by magnetic washer twice with 200 µL of assay buffer. Then to each well was added 50 µL of assay buffer containing 16 µg/mL solution of streptavidin-PE (Molecular Probes, Eugene, OR) and incubated for 45 min at room temperature, then washed twice by magnetic washer and resuspended in 100 µL of PBS. The plates were read on a Luminex-200 platform. For each bead 100 beads per well was collected. The median fluorescence intensity of beads was recorded for each bead and was used for analysis with the Bio-Plex Manager software (version 4.0; Bio-Rad, Hercules, CA, USA) using a 5P regression algorithm. A cytokine in samples was considered “detected” if its concentration was higher than the LLOD of the assay used and “not detected” when it fell below the LLOD.

### 2.10. Real-Time PCR

EVs from WT Jurkat and JinB8 T cells were prepared one day before the experiment and kept at −80 °C. The thawed EVs were immunocaptured as described above. HUVEC treated with CD63+ and MHC-I+ EVs were cultured for 48 h, and total RNA was prepared using the Trizol method. GSEA enrichment pathway analysis was used to validate mRNA expression of TNFα [18]. The first strand of cDNA was prepared using Maxima First Strand cDNA Synthesis Kit for RT-qPCR (Thermo scientific). A 4 μL volume of cDNA template was used along with primers for B2M, ß-actin, TNFα and ZEB1 as previously reported [11,12,18]. Primers for ZO-1-F-CGTTAGTCACCCAGGGCACAGG; ZO-1-R-GTATGTGGGCTGCTCGAGGT; ß-CATENIN-F-AAGTCTGGAGGCATTCCTGC; β-CATENIN-R-ACCAGCTAAACGCACTGCCA; SLUG-F-TGCACTGCGATGCCCAGTCT; SLUG-R-AAAACGCCTTGCCGCAGATC; SNAIL-F-CTTCCCGCAGGTTCCGCAGA; SNAIL-R-CTTCCCGCAGGTTCCGCAGA were from Integrated DNA Technologies.

The real-time PCR was performed using SYBR Green (Roche, Indianapolis, IN, USA) on Bio-Rad CT051073 model with amplification program; 94 °C for 10 min followed by 94 °C for 30 s, 55 °C for 30 followed by 39 cycles. The Gene Expression (ΛΛCT) was calculated relative to control as indicated. The significant value was calculated using Bio-Rad CFX Mastero software Version: 4.0.2325.0418.

### 2.11. Flow Cytometry Analysis

WT Jurkat and JinB8 T cells were stained with CD47-FITC, CD63-PECy7, and MHC-I-Alexa-647 conjugates and analyzed on a BD an LSR II System instrument (BD Biosciences, San Jose CA the data were subsequently analyzed and presented using FlowJO software (TreeStar, Ashland, OR, USA) as previously reported [11].

### 2.12. Negative Stain Electron Microscopy and Immunogold Labeling

Purified EVs, or EVs bound to MNPs were adhered to freshly glow discharged, formvar and carbon coated, 300-mesh gold grids (Electron Microscopy Sciences, Hatfield, PA, USA) by inverting grids on 5 µL drops of sample for 1 min and were rinsed by transferring the grids across 2 droplets of filtered PBS (Gibco). Grids were then rinsed twice on drops of distilled water before being placed on a drop of 2% aqueous uranyl acetate negative stain solution (Electron Microscopy Sciences) for 30 s, after which grids were blotted with filter paper.

To immunogold label EVs before negative staining, grids with adhered EVs were incubated on drops of blocking solution containing 2% BSA (Sigma, St. Louis, MO, USA) in PBS for 20 min. Primary antibody to CD63 (BioLegend, Clone H5C6) or MHC-I (BioLegend, Clone W6/32) was diluted to 20 µg/mL in blocking solution and grids were transferred to drops of primary antibody for 30 min. Then, grids were transferred across two drops of blocking solution for 20 min before incubation on drops of 10 nm gold-conjugated goat anti-mouse secondary antibody (Electron Microscopy Sciences) diluted 1:25 in blocking solution for 30 min. Finally, grids were rinsed with 3 drops of PBS and 2 drops of distilled water before negative staining. Grids were observed using a ThermoFisher Tecnai T20 transmission electron microscope operated at 200 kV, and images were acquired using an AMT NanoSprint1200 CMOS detector (Advanced Microscopy Techniques, Woburn, MA, USA).

### 2.13. NanoSight Analysis

EVs were purified using Exo-spin Exosome Purification with size exclusion chromatography (SEC) from Cell Guidance System according to the manufacturer’s instructions as described above in EV extraction section. A NanoSight NS300 was used for nanoparticle characterization according to manufacturer’s instructions as described previously [16].

### 2.14. Supplemental RNASEQ and Microarray Data

All data files are attached as supplemental Excel/text files.

## 3. Results

### 3.1. CD47 Regulates the Release of CD63^+^ and MHC-I^+^ EVs from Jurkat T Cells and Their RNA Contents

**Figure 1:**
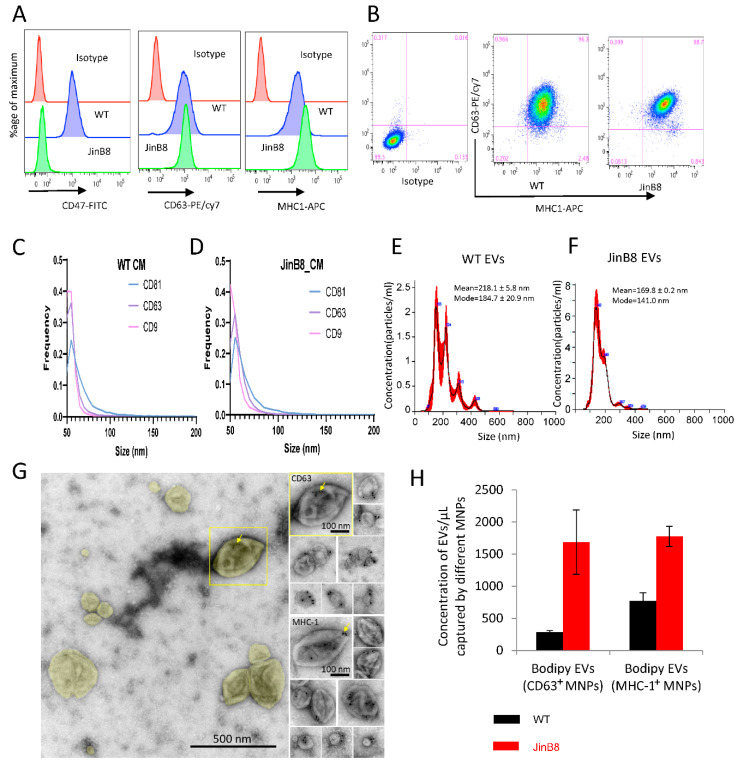
(**A**,**B**) WT Jurkat and JinB8 T cells were stained using conjugated CD47, CD63 and MHC-I antibodies and analyzed by flow cytometry. (**C**,**D**) NanoView analysis of size and expression of the tetraspanins CD9, CD63, and CD81 on EVs in concentrated conditioned media derived from WT Jurkat and JinB8 cells. (**E**,**F**) Size and concentration of EVs purified from WT and JinB8 cells were measured using Nano Sight. (**G**) Negative stain EM of immunogold labeled EVs purified from activated Jurkat T cells. **Left**, overview of EVs colorized yellow in a typical field of view. The boxed EV is immunogold labeled for CD63 and shown to the **upper right** in a montage with other CD63-labeled EVs. **Lower right**, a montage of EVs immunogold labeled for MHC-1. Arrows indicate 10-nm immunogold particles. (**H**) Quantification by flow cytometry of CD63^+^ and MHC-I^+^ EVs derived from WT Jurkat and JinB8 cells purified using size exclusion chromatography. Data are representative of two independents biological experiments.

Flow cytometry analysis demonstrated comparable surface expression of CD63 on WT Jurkat and JinB8 T cells, moderately elevated MHC-I levels on JinB8 T cells, and the expected absence of CD47 on JinB8 T cells (Figure 1A,B). The sizes of EVs released in conditioned media from WT Jurkat and JinB8 T cells bearing the EV-associated tetraspanins CD63, CD81, and CD9 were comparable based on NanoView analyses (Figure 1C,D), whereas NanoSight characterization of EVs purified using SEC indicated broader EV size distributions (Figure 1E,F). CD63 and MHC-I associated with EVs was also visualized by negative stain immunogold electron microscopy, which indicated a diverse size and morphologies of CD63^+^ and MHC-I^+^ EVs (Figure 1G).

Triggering threshold flow cytometry analysis was used to quantify the number of CD63^+^ and MHC-I^+^ EVs released from equal numbers of WT Jurkat and JinB8 T cells and captured using MNPs bearing the respective antibodies [13] (Figure 1H). JinB8 T cells released 6-fold more CD63^+^ and 2.2-fold more MHC-I^+^ EVs as compared to WT Jurkat T cells, which suggested that CD47 limits the biogenesis or release of these EV subsets by Jurkat T cells.

**Figure 2:**
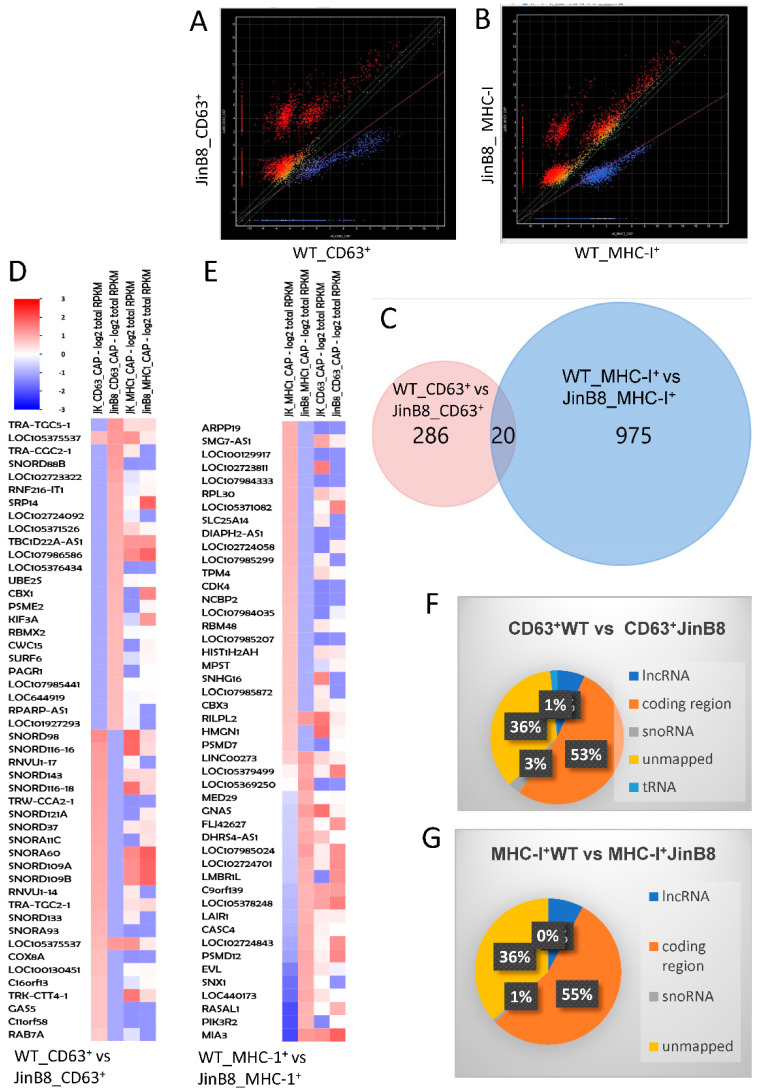
(**A**) Scatter graph showing abundance of RNAs in CD63^+^ EVs from WT Jurkat vs. JinB8 cells (red or blue = *p* < 0.05, n = 2, biological replicates). (**B**) Scatter graph showing abundance of RNAs in MHC-I^+^ EVs from WT Jurkat vs. JinB8 cells (*p* < 0.05, *n* = 2, biological replicates). (**C**). Venn diagram comparing 306 CD47-dependent annotated RNA transcripts in CD63^+^ EVs from WT Jurkat vs. JinB8 cells and 997 MHC-I^+^ EVs from WT Jurkat vs. JinB8 cells. (**D**,**E**) Heat maps with supervised clustering showing top CD47-dependent annotated RNA transcripts in CD63^+^ EVs from WT Jurkat vs. JinB8 cells ((**D**), *p* < 0.05) and in MHC-I^+^ EVs from WT Jurkat vs. JinB8 cells ((**E**), *p* < 0.05), respectively. (**F**,**G**) Pie charts showing classification of 306 CD47-dependent annotated RNA transcripts in CD63^+^ EVs from WT Jurkat vs. JinB8 cells ((**F**), *p* < 0.05) and 997 CD47-dependent transcripts in MHC-I^+^ EVs from WT Jurkat vs. JinB8 cells ((**G**), *p* < 0.05). Data files related with Figure 1; scatter graph cd63 pval05_Data_s1 and scatter graph cd63 pval05_Data_s2.

We previously reported the small RNA profiles of CD63^+^ and MHC-I^+^ EVs released from WT Jurkat T cells [13]. Small RNA seq analysis was used here to examine the CD47-dependence of the RNA transcripts in CD63^+^ and MHC-I^+^ EVs released from WT Jurkat and JinB8 T cells. Using alignment to the whole genome and analyzed as described earlier [13], 306 transcripts exhibited significant differential expression between CD63^+^ WT Jurkat and CD63^+^ JinB8 EVs (*p* < 0.05, Figure 2A), whereas 997 transcripts (995 mapped) exhibited differential expression between MHC-I^+^ WT Jurkat and JinB8 EVs (*p* < 0.05, Figure 2B). Remarkably, a Venn diagram analysis showed that only 20 CD47-dependent transcripts were shared between MHC-I^+^ and CD63^+^ EVs (Figure 2C). Many of the most CD47-dependent enriched or depleted transcripts in both EV types were noncoding RNAs (Figure 2D,E), although a global comparison revealed that a majority of the CD47-dependent transcripts represent protein coding mRNAs (Figure 2F,G).

### 3.2. Selective Uptake of MHC-I^+^ and CD63^+^ EVs by Target Endothelial Cells

**Figure 3:**
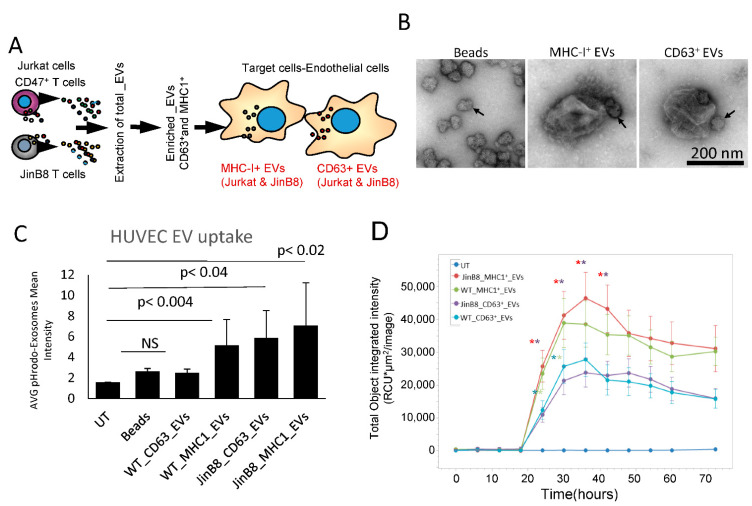
(**A**) Protocol for assessing HUVEC responses to MHC-I^+^ and CD63^+^ EVs from WT Jurkat vs. JinB8 cells. The respective EVs were pulled down using streptavidin magnetic nanobeads bearing CD63-biotin or MHC-I-biotin antibodies. (**B**) The nanobeads alone and captured EVs were visualized by negative stain EM. Arrows indicate nanobeads. (**C**) EVs labeled with pHrodo dye were captured, washed, and incubated with HUVEC for 24 h. HUVEC uptake was quantified using cell image Cytometry Celigo. (**D**) Kinetics of EV uptake by HUVEC were measured using Incucyte Live cell imaging analysis (*n* = 6 replicates per experiment, and two biological experiments). Significance was assessed using Student’s *t* test. For WT Jurkat CD63^+^ vs. WT Jurkat MHC-I^+^ EVs. Asterisk colors indicate significance of the corresponding line graphs (*) with *p* values significant at 24 h (0.03) and 30 h (0.04), and for JinB8 CD63^+^ vs. JinB8 MHC-I^+^ EVs from 24–42 h (0.0006, 0.002, 0.001, 0.002) respectively. No significant *p* values were observed between WT Jurkat MHC-I^+^ vs. JinB8 MHC-I^+^ EVs or WT Jurkat CD63^+^ versus JinB8 CD63^+^ EVs.

We examined the uptake of EVs into HUVEC and their effects on gene expression to compare the functional activities of CD63^+^ and MHC-I^+^ EVs released from WT Jurkat T cells (Figure 3A). CD63^+^ or MHC-I^+^ EVs extracted from WT Jurkat cells were immunocaptured with 50 nm streptavidin beads. Negative stain electron microscopy was used to confirm the presence of 200–300 nm diameter EVs with one or more beads attached in the captured samples, while control beads lacked associated EVs. (Figure 3B). Immunocaptured and labeled CD63^+^ and MHC-I^+^ EVs were added to HUVEC, and their uptake was measured using cell image cytometry (Celigo, Figure 3C). The results show that HUVEC take up more CD63^+^ and MHC-I^+^ EVs from JinB8 T cells than those from WT Jurkat T cells. The kinetics of EV uptake by HUVEC were evaluated by preparing pHRodo dye labeled EVs and measuring their uptake using Incucyte live cell image analysis (Figure 2D). As shown in Figure 3C, HUVEC take up more MHC-I^+^ EVs than CD63^+^ EVs from either WT Jurkat or JinB8 T cells. However, MHC-I^+^ EVs and CD63^+^ EVs showed the highest CD47-dependent uptake at ~36 h.

### 3.3. CD63^+^ and MHC-I^+^ EVs from WT Jurkat T Cells Regulate Different Molecular and Functional Programs in Recipient Endothelial Cells

**Figure 4:**
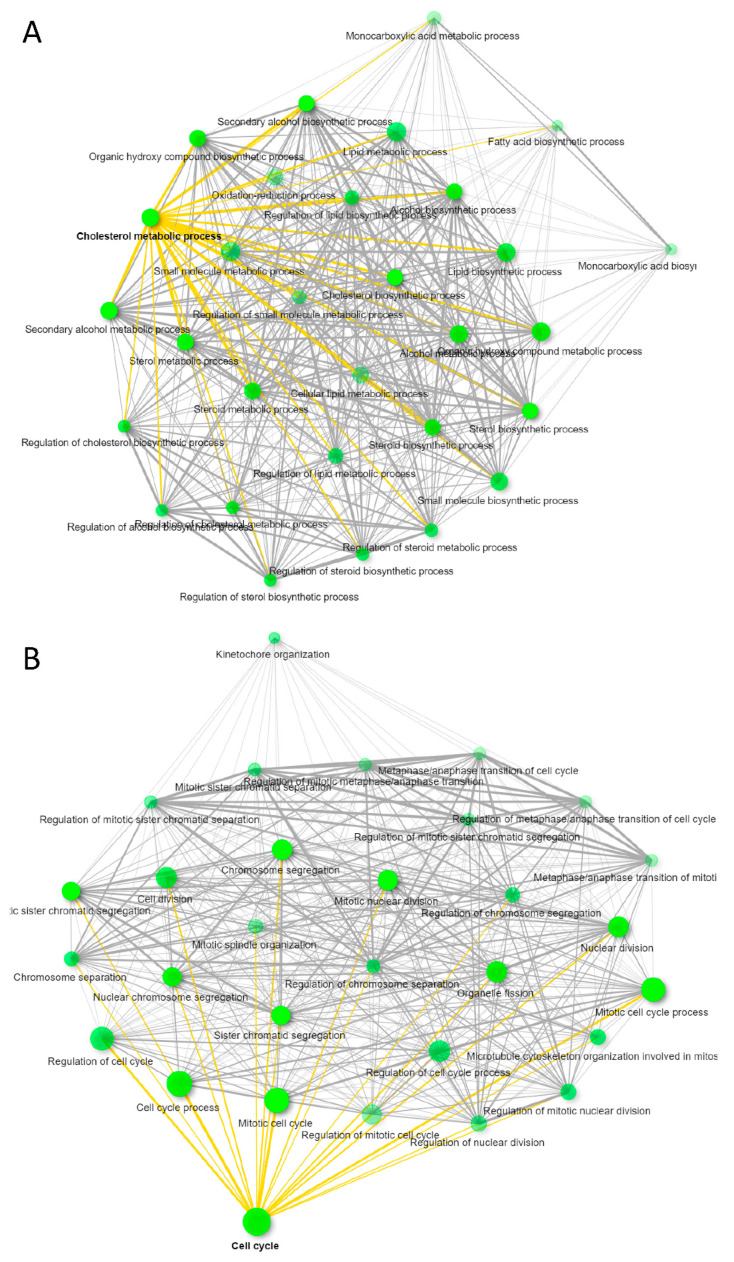
Immunocaptured CD63^+^ and MHC-I^+^ EVs released from WT Jurkat T cells were co-cultured with HUVEC for 3 days. Differential gene expression of HUVEC untreated vs. CD63^+^ EV or MHC-I^+^ EV treated HUVEC was determined using microarray analysis. (**A**) GO enrichment pathway network of untreated HUVEC vs. cells treated with WT Jurkat CD63^+^ EVs (>1.5 ± fold change, *p* ≤ 0.05). (**B**) GO enrichment pathway network of untreated HUVEC vs. cells treated with WT Jurkat MHC-I^+^ EVs (>1.5 ± fold change, *p* ≤ 0.05). Supplemental Materials Data files related with (**A**,**B**); Data/s2A, /Data/s2B, /Data/s2C.

To compare the functional effects of CD63^+^ and MHC-I^+^ EVs on recipient HUVEC, we treated HUVEC with the respective EVs complexed with nanobeads or control nanobeads alone for 3 days and then performed global transcriptome analyses on the RNA collected from the treated HUVEC cells. Treatment with CD63^+^ EVs changed expression of 235 HUVEC transcripts >1.5-fold with *p* < 0.05 as compared to untreated. Among those, 117 were upregulated and 118 downregulated as compared to untreated, but only 92 transcripts were annotated (Supplemental Materials Data s2A). ShinyGO v0.66 Gene Ontology Enrichment Analysis predicted most of the transcripts were related to lipid and cholesterol metabolism (Figure 4A). Similarly, treatment of HUVEC with MHC-I^+^ EVs showed 379 transcripts differentially expressed (304 downregulated and 76 upregulated > 1.5-fold, *p* < 0.05) as compared to untreated (Supplemental Materials Data s2B). Of these transcripts, 237 were annotated, and Gene Ontology Enrichment Analysis predicted that most of the genes were associated with cell cycle as shown in Figure 4B. In contrast, treatment of HUVEC with control beads significantly altered only 24 transcripts (>1.5-fold, *p* < 0.05), and no significant gene enrichment was observed (Supplemental Materials Data s2C). Therefore, CD63^+^ or MHC-I^+^ subsets of EVs produced by WT Jurkat T cells alter distinct transcriptional programs in target HUVEC.

MicroRNAs play a major role in the regulation of cholesterol metabolism in various cell types including endothelial cells [19]. Because our previous work identified significant differences in the miRNA contents of the CD63^+^ and MHC-I^+^ EVs [13], we looked for overlap between miRNAs known to regulate cholesterol metabolism and changes in the expression of their known target genes in HUVEC treated with CD63^+^ and MHC-I^+^ EVs (Table 1). *SREBF2* and *HMGCS1* are regulated by miR-185 and miR-miR-223, respectively [19]. These differed in CD63^+^ and MHC-I^+^ EVs, and treated HUVEC exhibited significant increases in SREBF2 and HMGCS1 mRNA only when treated with CD63^+^ EVs (Table 1). miR130b was present in CD63^+^ EVs but not detected in MHC-I^+^ EVs, and INSIG1 mRNA was induced 1,5-fold more in HUVEC following treatment with CD63^+^ compared to MHC-I^+^ EVs (Table 1). LDLR is a target of three miRNAs that we reported to differ between CD63^+^ and MHC-I^+^ EVs (miR-140, miR-185, miR-130b [13,19]. However, LDLR was induced approximately 2-fold in HUVEC treated with either EV subset (Table 1). Thus, some of the miRNAs that differ in CD63^+^ and MHC-I^+^ EVs are consistent with the differential regulation of their target genes by these EVs, but additional undefined noncoding RNAs that differ in these EV subsets and regulate cholesterol and lipid metabolism, including the selective induction of ACAT2 by CD63+ EVs (Table 1), and those in MHC-I^+^ EVs that selectively regulate cell cycle remain to be identified.

### 3.4. MHC-I^+^ EVs Upregulate More CD47-Dependent Transcripts in HUVEC Than CD63^+^ EVs

**Figure 5:**
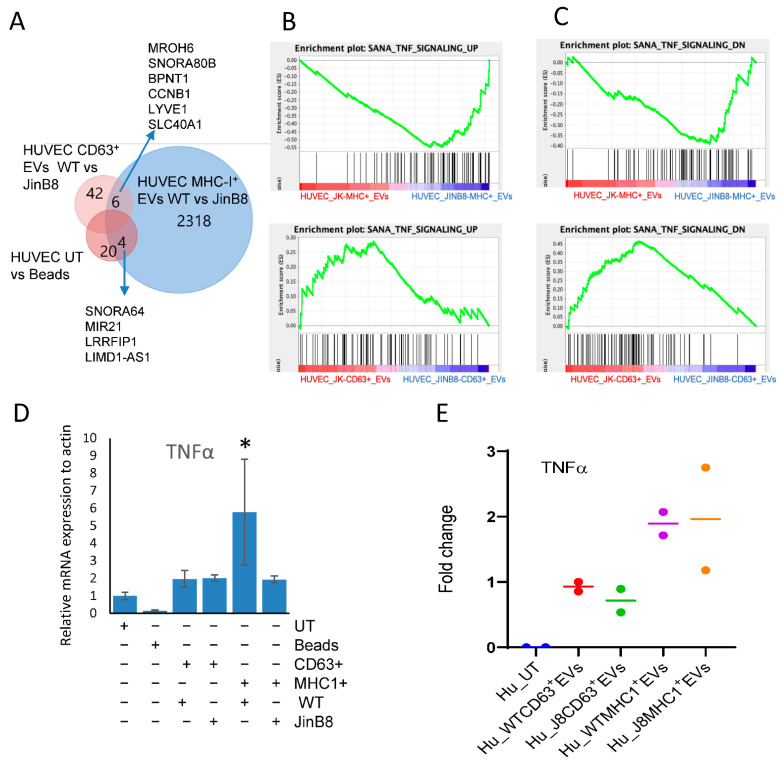
Immunocaptured CD63^+^ and MHC-I^+^ EVs as in Figure 3A were co-cultured with HUVEC for 3 days, and differential mRNA expression was evaluated using microarray analysis. (**A**) Venn diagram showing comparative analysis of CD47-dependent transcripts (*p* < 0.05) in HUVEC treated with beads alone or treated with CD63^+^ or MHC-I^+^ EVs from WT Jurkat or JinB8 T cells. (**B**,**C**) Geneset enrichment analysis plots showing enrichment of a TNF signaling signature in HUVEC treated with CD63^+^ EVs from WT Jurkat vs. JinB8 T cells (**B**) or MHC-I^+^ EVs from WT Jurkat vs. JinB8 T cells (**C**). (**D**) TNFα mRNA expression was validated via real-time PCR with ß-actin as control (1 biological replicate). (**E**) The increase in fold change of secreted TNFα in conditioned medium from HUVEC treated with the indicated EVs for 3 days. Secreted TNFα was undetectable from supernatant of untreated HUVEC cells, but control bead-treated sample had background values. Therefore, results are presented as fold change with respect to background value (*n* = 2). Supplemental Data files related with Figure 5; /Data/s3A, /Data/s3B, /Data/s2C. * *p* < 0.05.

To examine whether the functional effects of these EVs on target HUVEC depend on the status of CD47 in the source Jurkat T cells, we compared global transcripts after treating HUVEC with CD63^+^ and MHC-I^+^ EVs derived from WT Jurkat and JinB8 T cells. Treatment of HUVEC with WT Jurkat CD63^+^ EVs versus JinB8 CD63^+^ EVs resulted in differential up- or downregulation of 205 transcripts (>1.5-fold, *p* < 0.05). Among those, 113 transcripts were upregulated in HUVEC treated with JinB8 CD63+ EVs vs. WT Jurkat CD63^+^ EVs, and 87 transcripts were downregulated in HUVEC treated with JinB8 CD63^+^ EVs vs. WT Jurkat CD63^+^ EVs, but only 48 transcripts were mapped (Figure 5A and Figure S2A, Supplemental Materials Data s3A). The comparison of HUVEC treated with WT Jurkat MHC-I^+^ EVs vs. JinB8 MHC-I^+^ EVs led to total 3386 (2328 annotated) transcripts significantly with >1.5-fold (Supplemental Materials Data s3B). Among those, 2058 were upregulated in HUVEC treated with MHC-I^+^ JinB8 EVs, while 270 were upregulated in HUVEC treated with WT Jurkat MHC-I^+^ EVs, (Figure 5A and Figure S2B, Supplemental Materials Data s3B). This suggested that treatment of HUVEC with WT Jurkat MHC-I^+^ EVs versus JinB8 MHC-I^+^ EVs altered more transcripts than treatment with WT Jurkat CD63^+^ EVs versus JinB8 CD63^+^ EVs in CD47 dependent manner, which is also consistent with differential expressed transcripts from small RNA sequencing Figure 2B. We examined whether the CD47-dependent changes in treated HUVEC mRNA levels observed in Figure 5A were consistent with differences in levels of the same mRNAs in the respective EVs but found minimum overlap (data not shown). This suggests that noncoding RNAs in the EVs reprogrammed gene expression in the target HUVEC rather than direct transfer of the respective mRNAs from those EVs.

Gene enrichment analysis identified genes related to angiogenesis, VEGF and endothelial/mesenchymal transition (EMT) pathways that were altered between HUVEC treated with WT Jurkat CD63^+^ EVs and JinB8 CD63^+^ EVs or WT Jurkat MHC-I^+^ EVs and JinB8 MHC-I^+^ EVs in a CD47 dependent manner (data link in Supplementary Materials section). Treatment of HUVEC with either control beads, WT Jurkat CD63^+^ EVs, or JinB8 CD63^+^ EVs did not alter any EMT genes (Figure S3A,B). Treatment with MHC-I^+^ EVs from either WT Jurkat or JinB8 T cells altered the expression of SLUG significantly but not the other validated EMT genes examined (Figure S3C).

Genes that are upregulated in TNF signaling were also upregulated in HUVEC treated with MHC-I^+^ EVs from JinB8 as compared to WT Jurkat and upregulated in HUVEC treated with CD63^+^ EVs from WT Jurkat as compared to JinB8 cells. Inversely, genes that are downregulated in TNFα signaling were upregulated in HUVEC treated with MHC-I^+^ EVs from JinB8 as compared to WT Jurkat cells and downregulated in HUVEC treated with CD63^+^ EVs from WT Jurkat as compared to JinB8 cells. (Figure 5B,C). The TNFα pathway is a known CD47-dependent target of CD47 signaling in T cells [11,18]. To confirm regulation of this pathway in HUVEC, the cells were treated using MHC-I^+^ EVs and the respective depleted MHC-I^-^ EVs from WT Jurkat and JinB8 cells for 24–48 h (Figure S4A,B). HUVEC treated with WT Jurkat MHC-I^+^ EVs secreted more TNFα than untreated HUVEC or HUVEC treated with MHC-I^-^ EVs or JinB8 MHC-I^+^ EVs (Figure S4A). While secreted TNFα was undetectable at 48 h in untreated HUVEC. Increased secreted TNFα was consistently observed in HUVEC treated with MHC-I^+^ EVs after 24 h.

Treatment of HUVEC with MHC-I^+^ EVs or CD63^+^ EVs from WT Jurkat and JinB8 T cells for 3 days along with bead control demonstrated that TNFα mRNA expression was increased in WT Jurkat MHC-I^+^ EVs only as compared to all other treatments (Figure 5D). Similar results were found for secreted TNFα, which increased in HUVEC treated with MHC-I^+^ EVs from WT Jurkat and JinB8 cells as compared to HUVEC treated with the respective CD63^+^EVs (Figure 5E).

## 4. Discussion

We previously reported that CD47^+^, CD63^+^ and MHC-I^+^ subsets of EVs released by WT Jurkat T cells contain different populations of non-coding RNAs [13]. The present results demonstrate that both CD63^+^ and MHC-I^+^ subsets of EVs are taken up by HUVEC but elicit different patterns of gene expression in these recipient cells. We further identified several miRNAs enriched in CD63^+^ EVs that are known to regulate some of the mRNAs that were selectively induced in HUVEC treated with CD63^+^ EVs but not MHC-I^+^ EVs.

In addition to the observed functional differences between CD63^+^ and MHC-I^+^ EVs, some genes were identified that depend on whether these EV subsets were produced by WT Jurkat or CD47-deficient JinB8 T cells. Recently, we reported that CD47 regulates the trafficking to EVs of a subset of mRNAs and miRNAs bearing 7-methylguanosine (m7G)-modifications [16]. The patterns of CD47-dependent changes in HUVEC gene expression identified here provide a basis for future studies to identify specific m7G-modified RNAs that may account for the observed CD47-dependent changes in HUVEC gene expression induced by T cell EVs.

Lipid and cholesterol metabolism are emerging as important regulators of pathological angiogenesis associated with atherosclerosis and malignancy [20,21]. Two potential mechanisms could account for the differential effects of CD63^+^ and MHC-I^+^ EVs on cholesterol metabolism in recipient endothelial cells. Tetraspanins associate with cholesterol-rich microdomains, suggesting that effects of CD63^+^ EVs on cholesterol metabolism in recipient endothelial cells could result from an increased delivery of cholesterol by CD63^+^ EVs. Alternatively, noncoding RNAs that regulate expression of genes involved in cholesterol metabolism could be enriched in CD63^+^ EVs. The latter is consistent with the enrichment of miR-20b, a regulator of ABCA1 that controls cholesterol efflux in several cell types by facilitating the export of cellular cholesterol to its extracellular acceptor protein apolipoprotein-A1 [19] in CD63^+^ versus MHC-I^+^ EVs released by WT Jurkat cells [13]. ABCA1 is also regulated by miR-106b, which is differentially released in CD63^+^ versus MHC-I^+^ EVs [13]. Another regulator of cholesterol homeostasis is miRNA-185 [19], which is differentially released into CD63^+^ versus MHC-I^+^ EVs [13]. Hsa-let-7d, which is enriched in CD63^+^ versus CD63^−^ EVs [13], was identified as a regulator of cholesterol biosynthesis [22]. Although the functions of these specific EV miRNAs remain to be confirmed in the treated endothelial cells, several of the mi-RNAs previously found to be differentially enriched in CD63^+^ EVs are known regulators of cholesterol homeostasis.

The uptake efficiency of CD63^+^ and MHC-I^+^ EV subsets differed in target HUVEC and may also be CD47 dependent. The preferential uptake of MHC-I^+^ EVs over CD63^+^ EVs may be due to interaction between HUVEC and T cell-derived EVs as has shown in endothelial and immune cells [23] or CD47 independent interactions of MHC-I with its inhibitory counter-receptor LILRB1 [24]. The mechanisms by which CD47 regulates EV uptake remain to be defined, but one possibility involves engaging its counter-receptor SIRPα, which is expressed on HUVEC [12]. Alternatively, lateral interactions of CD47 with integrins on EVs could regulate uptake mediated by the respective integrin ligands such as VCAM1 on endothelial cells.

Our data indicate that secreted TNFα is specifically induced by MHC-I^+^ Jurkat T cell EVs. The functions of TNFα include regulating the EMT transition in many cancers [25,26], These pathways are interrelated and are elevated via subsets of MHC-I^+^ EVs in a CD47-dependent manner. Expression of mRNAs encoding additional cytokines, chemokines, and their receptors including CXCL10, CXCL11, CXCL8, IL1A, IL1RAP, IL36RN and IL9R in HUVEC were differentially regulated by WT Jurkat versus JinB8 T cell MHC-I^+^ EVs. These cytokines and chemokines may in turn mediate some of the effects of these EVs on HUVEC gene expression.

Small RNA sequencing analysis showed that MHC-I^+^ EVs produced by JinB8 T cells versus WT Jurkat T cells have more differentially expressed transcripts than CD63^+^ EVs produced by the two cell types. This is consistent with the higher number of differentially expressed genes in HUVEC treated with MHC-I^+^ EVs from JinB8 versus WT Jurkat T cells as compared to HUVEC treated with CD63^+^ EVs from the two cell types. Because there was no substantial overlap between mRNAs differing in the EVs and the transcripts in HUVEC altered by treatment with these EVs, the direct transfer to EV mRNAs to target HUVEC is not a major mechanism, and non-coding RNAs may play the major regulatory role in reprogramming of target cells. We identified several such miRNAs in the EV subsets that are consistent with effects of the respective EVs on expression of mRNAs involved in lipid and cholesterol metabolism in HUVEC, but additional miRNAs mediating the other observed transcriptional responses in treated HUVEC or other indirect mechanisms remain to be identified.

Future studies will focus on identifying mechanisms by which CD63 and MHC-I regulate EV uptake and the distinct functional effects of MHC-I^+^ EVs and CD63^+^ EVs on endothelial cells and other types of target cells. These studies may identify additional functions of EVs produced by T cells that regulate physiological angiogenic and inflammatory pathways and antitumor immunity.

## Figures and Tables

**Table 1:** Genes involved in cholesterol and lipid metabolism in HUVEC that are differentially regulated by CD63^+^ vs. MHC-I^+^ EVs and miRNAs known to regulate these genes that differ in CD63^+^ and MHC-I^+^ EVs released by WT Jurkat T cells.

Gene	Function ^1^	CD63^+^ EV vs. UT (Fold)	*p* Value	MHC-I^+^ EV vs. UT (Fold)	*p* Value	miRNAs Differing in CD63^+^ vs. MHC-I^+^ EVs ^2^
*LDLR*	cholesterol uptake	2.0	2.8 × 10^−6^	1.93	5.2 × 10^−6^	miR-140, miR-185, miR-130b
*SREBF2*	cholesterol biosynthesis	1.5	0.00032		NS	miR-185
*INSIG1*	cholesterol biosynthesis	2.81	2.6 × 10^−5^	1.97	0.00124	miR-130b
*HMGCR*	cholesterol biosynthesis	2.1	0.032		NS	
*HMGCS1*	cholesterol biosynthesis	2.99	0.0052		NS	miR-223
*ACAT2*	lipid/cholesterol metabolism	2.3	0.014		NS	

^1^ Data for target genes of the listed miRNAs are from [19]. NS, not significant. ^2^ Differential export of the miRNAs in CD63^+^ and MHC-I^+^ EVs released by WT Jurkat T cells is from [13].

## Data Availability

Data files are attached as Supplemental Files.

## Supplementary Materials

The following are available online at https://www.mdpi.com/article/10.3390/biomedicines9111705/s1, (Scatter graph cd63 pval05), /Data/s2 (Scatter graph MHC1 pval05), /Data/s2A (Untreated vs JK-CD63_corrected_100521_Data_s2A), /Data/s2B (Untreated vs JK-MHC_corrected_1.5_ Data_s2B), /Data/s2C (Untreated vs Beads_corrected_100521_ Data_s2C), /Data/s3A (HuVec CD63 JK_vs_Jinb8 1.5FC_100521_Data s3A), /Data/s3B (JinB8-MHC vs JK-MHC ANOVA_100521_Supplemental Data s3B). Figures S1–S4: (Biomedicine_Supplementary Figures). HTML links for complete gsea_report_for_HUVEC treated with MHC-I+ EVs and CD63+ EVs from WT(JK) and JinB8T cells. (1) Huvec_JK_MHC-Exo_1539178433246, (2) gsea_report_for_Huvec_JinB8_MHC-Exo_1539178433246; (3) gsea_report_for_Huvec_JK_CD63-Exo_1539178329650, (4) gsea_report_for_Huvec_JINB8_CD63-Exo_1539178329650.
