# Supplementary material for: CD63+ and MHC Class I+ Subsets of Extracellular Vesicles Produced by Wild-Type and CD47-Deficient Jurkat T Cells Have Divergent Functional Effects on Endothelial Cell Gene Expression
Source: Biomedicines. 2021 Nov 17;9(11):1705. doi: 10.3390/biomedicines9111705 (PMC8615535; doi:10.3390/biomedicines9111705)

Supplementary Figures:

Figure S1. A) RNAs enriched in CD63$^+$ and MHC-I$^+$ EVs derived from WT Jurkat and JinB8 T cells. log2 total RPKM values show 618 transcripts present in CD63$^+$ WT EVs, while 1314 in CD63$^+$ JinB8 EVs respectively. Venn diagram showing overlap of 278 transcripts among total transcripts. B) log2 total RPKM values show 535 transcripts mapped in HUVEC treated with MHC-I$^+$ WT Jurkat EVs while 1414 in cells treated with MHC-I$^+$ JinB8 EVs, respectively. Venn diagram showing overlap of 384 transcripts among total transcripts. C&D) Types of RNAs between treatments with WT Jurkat CD63$^+$ vs JinB8 CD63$^+$ and WT Jurkat MHC-I$^+$ vs JinB8 MHC-I$^+$ based on small RNA data analysis aligned to the whole genome.

FigureS1

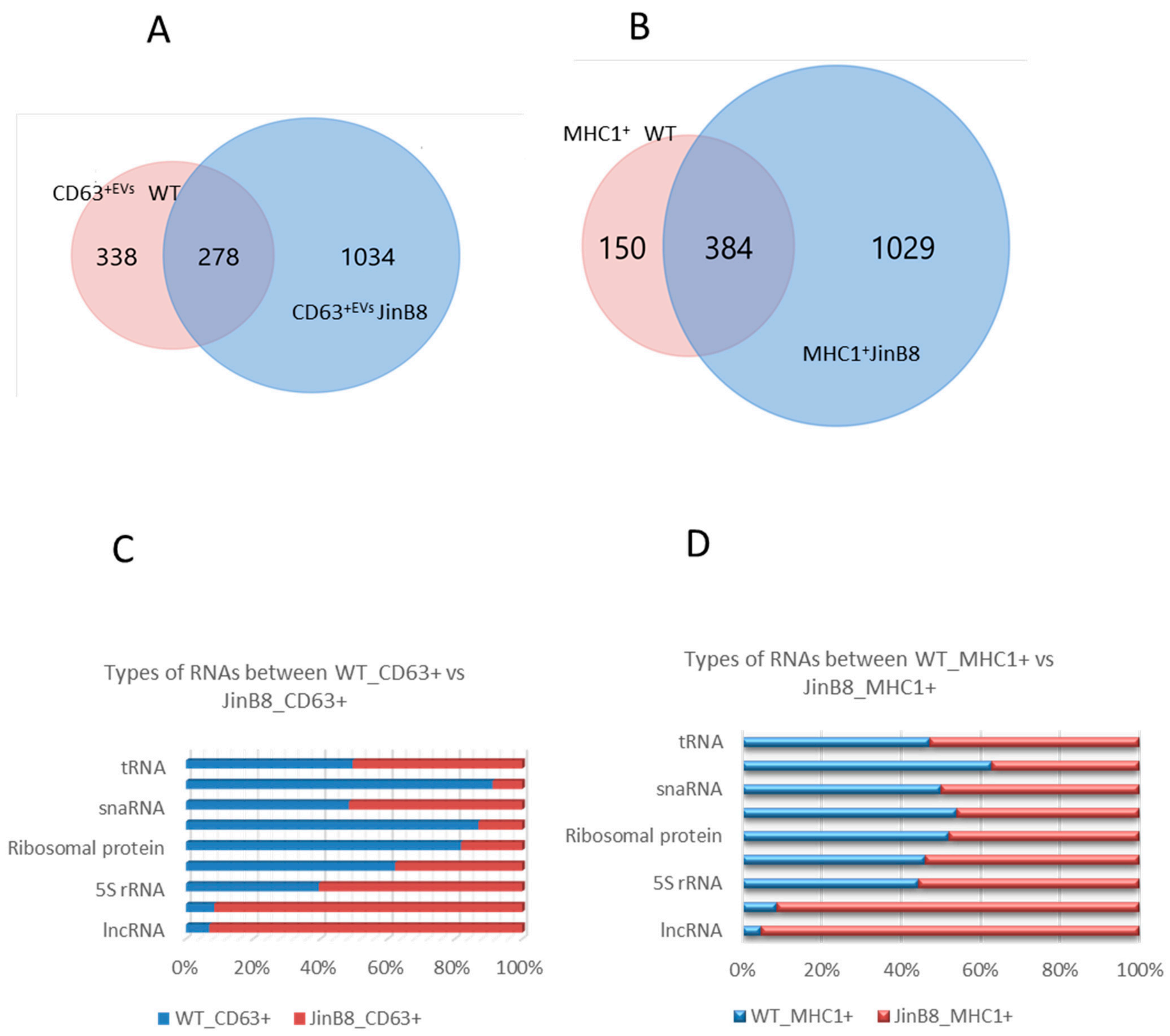

Figure S2. A&B) Hierarchical clustered of differentially expressed mRNA between HUVEC cells treated with WT Jurkat-derived MHC-I$^+$ and JinB8-derived MHC-I$^+$ EVs or CD63+ and JinB8-derived CD63$^+$ EVs based on microarray analysis of HUVEC.

Figure S2

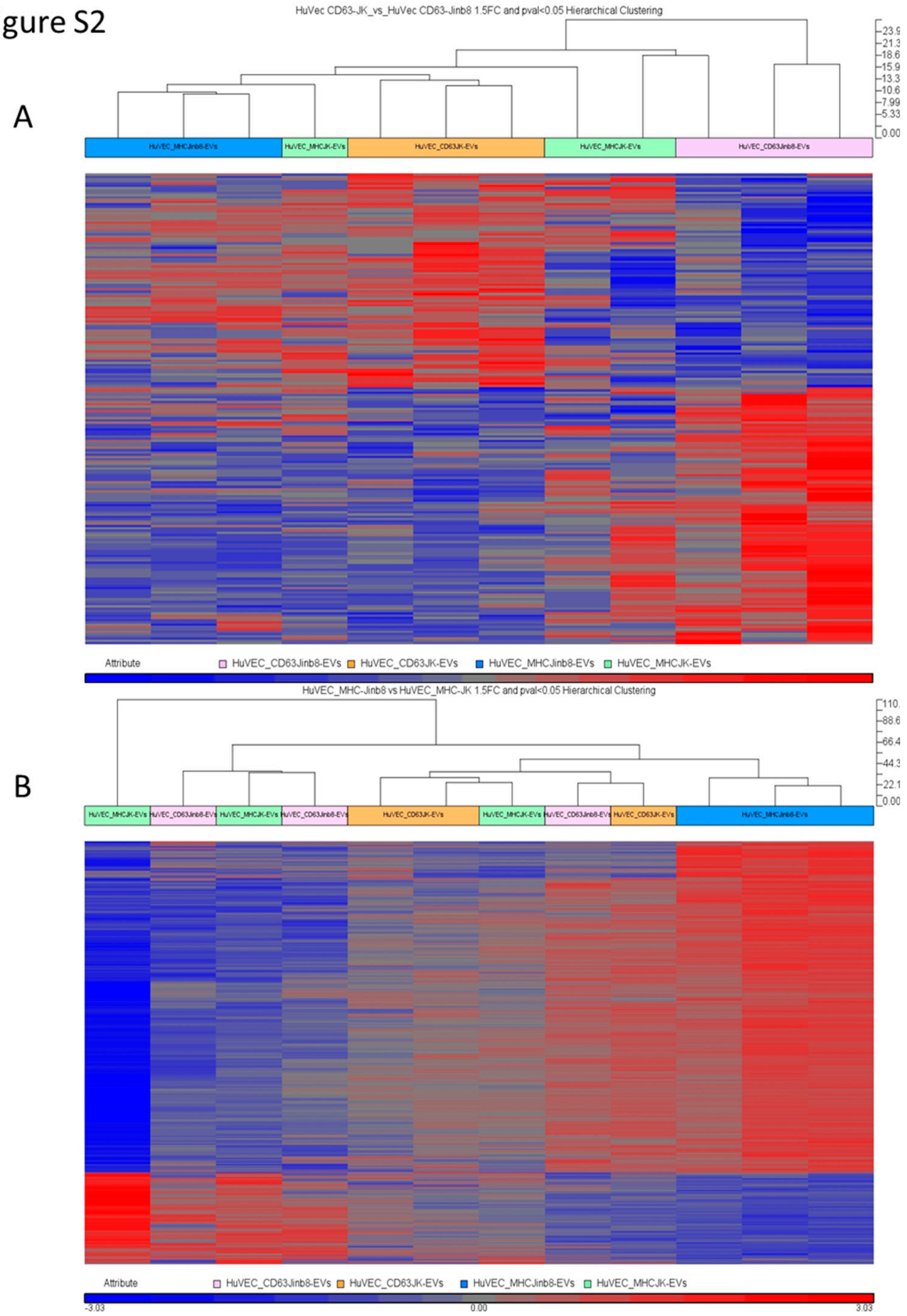

Figure S3. Validation of GSEA enrichment for EMT pathway. CD63⁺ and MHC-I⁺ EVs were extracted from WT Jurkat and JinB8 T cells and co-cultured with HUVEC for 3 days using beads as a control. mRNA expression of CTNNB1 (ß-CATENIN), ZO-1, ZEB1, SLUG, SNAIL was analyzed using actin as control. A) Untreated vs Beads. B) WT Jurkat CD63⁺ vs JinB8 CD63⁺ and C) WT Jurkat MHC-I⁺ vs JinB8 MHC-I⁺.

Figure S3

A

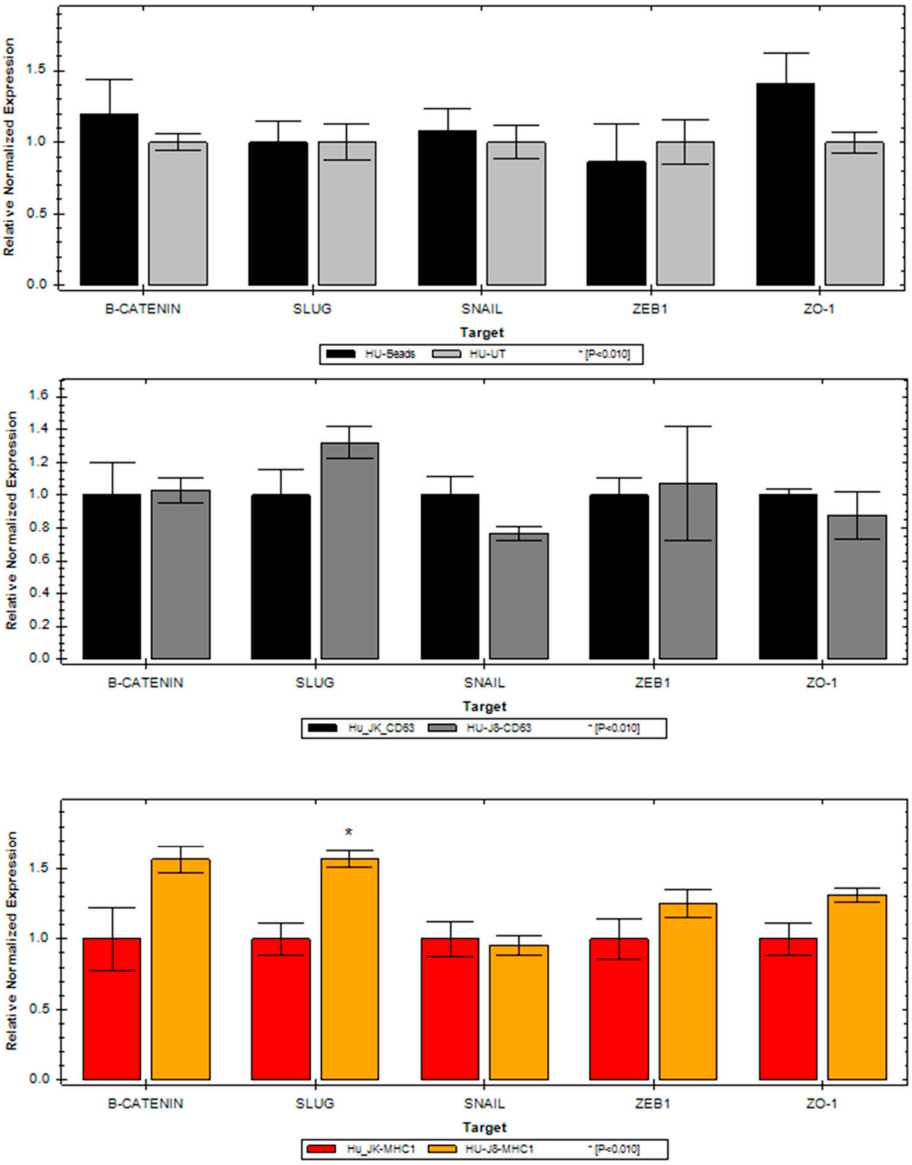

Figure S4. A&B) Validation of secreted TNFα in HUVEC treated with captured MHC-I^{+} and depleted MHC-I^{-} EVs from WT Jurkat and JinB8 T cells and co-cultured with HUVEC for 24-48 h.

Figure S4

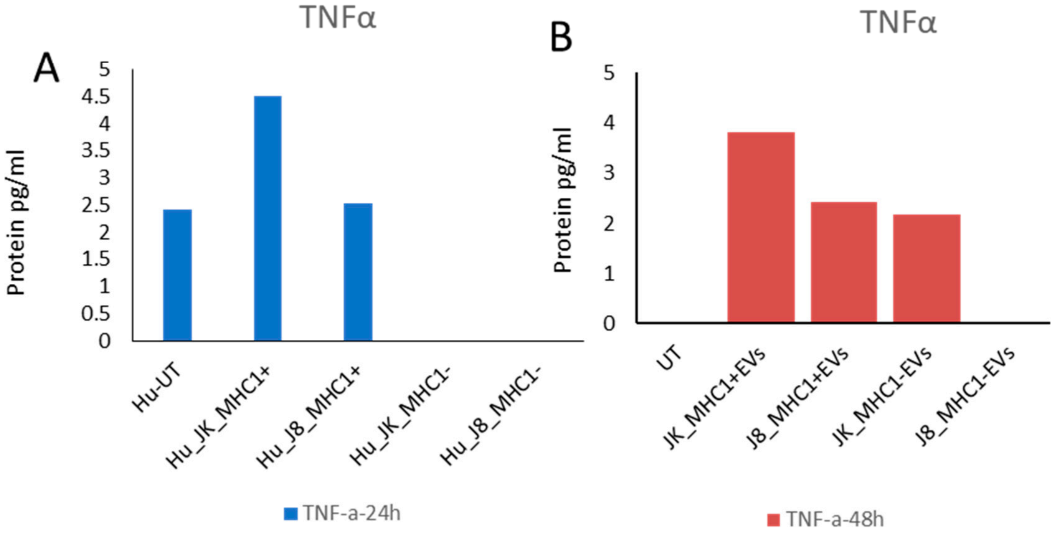

Supplement: Supplementary file 1 [file biomedicines-09-01705-s001.zip › Biomedicine_Supplementary Figures.pdf]
