# Supplementary material for: CD63+ and MHC Class I+ Subsets of Extracellular Vesicles Produced by Wild-Type and CD47-Deficient Jurkat T Cells Have Divergent Functional Effects on Endothelial Cell Gene Expression
Source: Biomedicines. 2021 Nov 17;9(11):1705. doi: 10.3390/biomedicines9111705 (PMC8615535; doi:10.3390/biomedicines9111705)
Supplement: Supplementary file 1 [file biomedicines-09-01705-s001.zip › gsea_report_for_Huvec_JINB8_CD63-Exo_1539178329650.html]

Report for Huvec\_JINB8\_CD63-Exo 1539178329650 [GSEA]

| GS  follow link to MSigDB | GS DETAILS | SIZE | ES | NES | NOM p-val | FDR q-val | FWER p-val | RANK AT MAX | LEADING EDGE || 1 | CHIANG\_LIVER\_CANCER\_SUBCLASS\_INTERFERON\_UP | Details ... | 26 | -0.45 | -1.44 | 0.000 | 0.734 | 0.538 | 6199 | tags=50%, list=30%, signal=72% |
| 2 | GO\_NEGATIVE\_REGULATION\_OF\_EPITHELIAL\_TO\_MESENCHYMAL\_TRANSITION | Details ... | 22 | -0.34 | -1.35 | 0.000 | 1.000 | 0.902 | 5801 | tags=45%, list=28%, signal=63% |
| 3 | BEGUM\_TARGETS\_OF\_PAX3\_FOXO1\_FUSION\_DN | Details ... | 45 | -0.28 | -1.25 | 0.078 | 1.000 | 1.000 | 3678 | tags=22%, list=18%, signal=27% |
| 4 | GSE3920\_IFNA\_VS\_IFNB\_TREATED\_ENDOTHELIAL\_CELL\_DN | Details ... | 157 | -0.24 | -1.24 | 0.076 | 1.000 | 1.000 | 4324 | tags=24%, list=21%, signal=30% |
| 5 | CHIANG\_LIVER\_CANCER\_SUBCLASS\_POLYSOMY7\_UP | Details ... | 74 | -0.38 | -1.20 | 0.163 | 1.000 | 1.000 | 4615 | tags=42%, list=23%, signal=54% |
| 6 | GSE13485\_DAY1\_VS\_DAY3\_YF17D\_VACCINE\_PBMC\_UP | Details ... | 158 | -0.42 | -1.19 | 0.214 | 1.000 | 1.000 | 5504 | tags=45%, list=27%, signal=61% |
| 7 | PIGF\_UP.V1\_DN | Details ... | 182 | -0.40 | -1.18 | 0.343 | 1.000 | 1.000 | 4485 | tags=39%, list=22%, signal=50% |
| 8 | GSE13485\_CTRL\_VS\_DAY1\_YF17D\_VACCINE\_PBMC\_DN | Details ... | 143 | -0.42 | -1.18 | 0.286 | 1.000 | 1.000 | 5082 | tags=43%, list=25%, signal=57% |
| 9 | GSE13485\_DAY3\_VS\_DAY21\_YF17D\_VACCINE\_PBMC\_DN | Details ... | 150 | -0.43 | -1.16 | 0.417 | 1.000 | 1.000 | 5815 | tags=48%, list=28%, signal=67% |
| 10 | GSE13485\_DAY1\_VS\_DAY7\_YF17D\_VACCINE\_PBMC\_UP | Details ... | 158 | -0.42 | -1.15 | 0.417 | 0.974 | 1.000 | 5519 | tags=45%, list=27%, signal=61% |
| 11 | GSE13485\_DAY7\_VS\_DAY21\_YF17D\_VACCINE\_PBMC\_DN | Details ... | 152 | -0.40 | -1.14 | 0.092 | 0.931 | 1.000 | 5884 | tags=45%, list=29%, signal=62% |
| 12 | CHIANG\_LIVER\_CANCER\_SUBCLASS\_INTERFERON\_DN | Details ... | 45 | -0.32 | -1.13 | 0.186 | 0.912 | 1.000 | 5071 | tags=40%, list=25%, signal=53% |
| 13 | VEGF\_A\_UP.V1\_UP | Details ... | 188 | -0.28 | -1.13 | 0.132 | 0.847 | 1.000 | 4286 | tags=24%, list=21%, signal=30% |
| 14 | JAEGER\_METASTASIS\_DN | Details ... | 253 | -0.38 | -1.12 | 0.417 | 0.797 | 1.000 | 4860 | tags=34%, list=24%, signal=44% |
| 15 | GSE13485\_CTRL\_VS\_DAY21\_YF17D\_VACCINE\_PBMC\_DN | Details ... | 147 | -0.32 | -1.11 | 0.076 | 0.783 | 1.000 | 5569 | tags=39%, list=27%, signal=53% |
| 16 | GSE3920\_UNTREATED\_VS\_IFNG\_TREATED\_ENDOTHELIAL\_CELL\_UP | Details ... | 147 | -0.23 | -1.07 | 0.187 | 0.906 | 1.000 | 3496 | tags=17%, list=17%, signal=20% |
| 17 | CHIANG\_LIVER\_CANCER\_SUBCLASS\_PROLIFERATION\_DN | Details ... | 173 | -0.33 | -1.07 | 0.272 | 0.862 | 1.000 | 5562 | tags=43%, list=27%, signal=58% |
| 18 | GSE4748\_LPS\_VS\_LPS\_AND\_CYANOBACTERIUM\_LPSLIKE\_STIM\_DC\_3H\_DN | Details ... | 150 | -0.30 | -1.07 | 0.421 | 0.824 | 1.000 | 3163 | tags=23%, list=15%, signal=27% |
| 19 | GSE13485\_CTRL\_VS\_DAY3\_YF17D\_VACCINE\_PBMC\_UP | Details ... | 153 | -0.24 | -1.06 | 0.259 | 0.789 | 1.000 | 5637 | tags=34%, list=28%, signal=47% |
| 20 | GO\_EPITHELIAL\_TO\_MESENCHYMAL\_TRANSITION | Details ... | 56 | -0.26 | -1.01 | 0.435 | 0.877 | 1.000 | 5107 | tags=30%, list=25%, signal=40% |
| 21 | GSE4748\_CYANOBACTERIUM\_LPSLIKE\_VS\_LPS\_AND\_CYANOBACTERIUM\_LPSLIKE\_STIM\_DC\_3H\_DN |  | 168 | -0.30 | -1.01 | 0.512 | 0.841 | 1.000 | 4296 | tags=25%, list=21%, signal=31% |
| 22 | CHIANG\_LIVER\_CANCER\_SUBCLASS\_CTNNB1\_DN |  | 163 | -0.26 | -0.98 | 0.468 | 0.860 | 1.000 | 4662 | tags=28%, list=23%, signal=36% |
| 23 | CHIANG\_LIVER\_CANCER\_SUBCLASS\_CTNNB1\_UP |  | 168 | -0.27 | -0.98 | 0.288 | 0.827 | 1.000 | 4741 | tags=30%, list=23%, signal=39% |
| 24 | AIGNER\_ZEB1\_TARGETS |  | 33 | -0.39 | -0.97 | 0.599 | 0.817 | 1.000 | 3098 | tags=33%, list=15%, signal=39% |
| 25 | HOSHIDA\_LIVER\_CANCER\_LATE\_RECURRENCE\_DN |  | 68 | -0.21 | -0.93 | 0.602 | 0.863 | 1.000 | 2998 | tags=18%, list=15%, signal=21% |
| 26 | GO\_REGULATION\_OF\_EPITHELIAL\_TO\_MESENCHYMAL\_TRANSITION |  | 65 | -0.17 | -0.93 | 0.588 | 0.832 | 1.000 | 5801 | tags=26%, list=28%, signal=36% |
| 27 | GO\_CARDIAC\_EPITHELIAL\_TO\_MESENCHYMAL\_TRANSITION |  | 24 | -0.20 | -0.86 | 0.624 | 0.956 | 1.000 | 3915 | tags=21%, list=19%, signal=26% |
| 28 | GSE13485\_DAY3\_VS\_DAY7\_YF17D\_VACCINE\_PBMC\_UP |  | 157 | -0.17 | -0.85 | 0.567 | 0.937 | 1.000 | 5239 | tags=25%, list=26%, signal=33% |
| 29 | GSE4748\_CTRL\_VS\_LPS\_STIM\_DC\_3H\_DN |  | 188 | -0.14 | -0.84 | 0.906 | 0.908 | 1.000 | 2180 | tags=9%, list=11%, signal=9% |
| 30 | GSE17708\_A549\_TGFB\_0.5HRS\_UP |  | 373 | -0.14 | -0.81 | 0.600 | 0.931 | 1.000 | 5628 | tags=24%, list=28%, signal=32% |
| 31 | HOSHIDA\_LIVER\_CANCER\_SURVIVAL\_UP |  | 73 | -0.16 | -0.80 | 0.777 | 0.916 | 1.000 | 1355 | tags=7%, list=7%, signal=7% |
| 32 | HOSHIDA\_LIVER\_CANCER\_LATE\_RECURRENCE\_UP |  | 57 | -0.17 | -0.79 | 0.907 | 0.904 | 1.000 | 1709 | tags=9%, list=8%, signal=10% |
| 33 | GSE13485\_CTRL\_VS\_DAY7\_YF17D\_VACCINE\_PBMC\_UP |  | 163 | -0.14 | -0.69 | 0.714 | 0.978 | 1.000 | 5139 | tags=21%, list=25%, signal=28% |
| 34 | HOSHIDA\_LIVER\_CANCER\_SURVIVAL\_DN |  | 110 | -0.17 | -0.69 | 0.825 | 0.951 | 1.000 | 4173 | tags=23%, list=20%, signal=28% |
| 35 | THUM\_MIR21\_TARGETS\_HEART\_DISEASE\_UP |  | 16 | -0.24 | -0.67 | 0.832 | 0.937 | 1.000 | 5121 | tags=31%, list=25%, signal=42% |
| 36 | GROSS\_HIF1A\_TARGETS\_DN |  | 24 | -0.20 | -0.58 | 0.904 | 0.979 | 1.000 | 2109 | tags=13%, list=10%, signal=14% |
| 37 | GO\_MESENCHYMAL\_TO\_EPITHELIAL\_TRANSITION |  | 15 | -0.26 | -0.50 | 0.908 | 0.979 | 1.000 | 6685 | tags=53%, list=33%, signal=79% |
Table: Gene sets enriched in phenotype **Huvec\_JINB8\_CD63-Exo (3 samples)**[plain text format]****

  
