# Supplementary material for: CD63+ and MHC Class I+ Subsets of Extracellular Vesicles Produced by Wild-Type and CD47-Deficient Jurkat T Cells Have Divergent Functional Effects on Endothelial Cell Gene Expression
Source: Biomedicines. 2021 Nov 17;9(11):1705. doi: 10.3390/biomedicines9111705 (PMC8615535; doi:10.3390/biomedicines9111705)
Supplement: Supplementary file 1 [file biomedicines-09-01705-s001.zip › gsea_report_for_Huvec_JINB8_MHC-Exo_1539178433246.html]

Report for Huvec\_JINB8\_MHC-Exo 1539178433246 [GSEA]

| GS  follow link to MSigDB | GS DETAILS | SIZE | ES | NES | NOM p-val | FDR q-val | FWER p-val | RANK AT MAX | LEADING EDGE || 1 | GSE4748\_CTRL\_VS\_CYANOBACTERIUM\_LPSLIKE\_STIM\_DC\_3H\_DN | Details ... | 192 | -0.51 | -1.44 | 0.000 | 1.000 | 0.348 | 6093 | tags=55%, list=30%, signal=78% |
| 2 | GSE14415\_INDUCED\_TREG\_VS\_TCONV\_UP | Details ... | 173 | -0.41 | -1.42 | 0.000 | 0.548 | 0.390 | 4490 | tags=35%, list=22%, signal=45% |
| 3 | GSE36476\_CTRL\_VS\_TSST\_ACT\_40H\_MEMORY\_CD4\_TCELL\_YOUNG\_DN | Details ... | 194 | -0.41 | -1.34 | 0.214 | 0.808 | 0.711 | 4792 | tags=38%, list=23%, signal=49% |
| 4 | GSE22886\_CTRL\_VS\_LPS\_24H\_DC\_DN | Details ... | 188 | -0.50 | -1.34 | 0.000 | 0.652 | 0.711 | 5512 | tags=48%, list=27%, signal=65% |
| 5 | GROSS\_HYPOXIA\_VIA\_ELK3\_ONLY\_UP | Details ... | 33 | -0.41 | -1.32 | 0.187 | 0.570 | 0.711 | 4741 | tags=42%, list=23%, signal=55% |
| 6 | GSE31019\_SKOV3\_INF-6HRSUDOWNVSCTRL | Details ... | 88 | -0.35 | -1.29 | 0.000 | 0.643 | 0.711 | 4152 | tags=36%, list=20%, signal=45% |
| 7 | GROSS\_HYPOXIA\_VIA\_ELK3\_AND\_HIF1A\_UP | Details ... | 138 | -0.46 | -1.28 | 0.000 | 0.601 | 0.711 | 6306 | tags=57%, list=31%, signal=81% |
| 8 | CHIANG\_LIVER\_CANCER\_SUBCLASS\_UNANNOTATED\_DN | Details ... | 184 | -0.67 | -1.27 | 0.000 | 0.564 | 0.711 | 5288 | tags=72%, list=26%, signal=97% |
| 9 | VANOEVELEN\_MYOGENESIS\_SIN3A\_TARGETS | Details ... | 209 | -0.44 | -1.27 | 0.091 | 0.518 | 0.711 | 5568 | tags=47%, list=27%, signal=64% |
| 10 | MTOR\_UP.N4.V1\_UP | Details ... | 191 | -0.45 | -1.27 | 0.000 | 0.470 | 0.711 | 4485 | tags=45%, list=22%, signal=56% |
| 11 | SANA\_TNF\_SIGNALING\_UP | Details ... | 78 | -0.55 | -1.27 | 0.000 | 0.431 | 0.711 | 5050 | tags=51%, list=25%, signal=68% |
| 12 | GSE3920\_IFNB\_VS\_IFNG\_TREATED\_ENDOTHELIAL\_CELL\_UP | Details ... | 158 | -0.30 | -1.27 | 0.000 | 0.399 | 0.711 | 3994 | tags=27%, list=20%, signal=34% |
| 13 | GSE17708\_A549\_TGFB\_24HRS\_DOWN | Details ... | 303 | -0.36 | -1.26 | 0.000 | 0.418 | 0.711 | 5955 | tags=47%, list=29%, signal=65% |
| 14 | GSE13485\_DAY1\_VS\_DAY21\_YF17D\_VACCINE\_PBMC\_DN | Details ... | 177 | -0.73 | -1.25 | 0.000 | 0.398 | 0.711 | 3509 | tags=71%, list=17%, signal=85% |
| 15 | GSE17708\_A549\_TGFB\_72HRS\_DOWN | Details ... | 299 | -0.30 | -1.25 | 0.000 | 0.383 | 0.711 | 5531 | tags=38%, list=27%, signal=51% |
| 16 | GSE4748\_CTRL\_VS\_LPS\_STIM\_DC\_3H\_DN | Details ... | 188 | -0.26 | -1.24 | 0.000 | 0.403 | 0.711 | 4925 | tags=31%, list=24%, signal=40% |
| 17 | GSE49329\_MURINE MICROGLIA\_IL4\_DOWN | Details ... | 127 | -0.24 | -1.24 | 0.077 | 0.382 | 0.711 | 5843 | tags=37%, list=29%, signal=51% |
| 18 | GSE31019\_HT1080\_INF-12HRSUPVSCTRL | Details ... | 76 | -0.61 | -1.24 | 0.000 | 0.368 | 0.711 | 6249 | tags=68%, list=31%, signal=98% |
| 19 | HU\_ANGIOGENESIS\_DN | Details ... | 37 | -0.61 | -1.24 | 0.000 | 0.351 | 0.711 | 6117 | tags=70%, list=30%, signal=100% |
| 20 | GSE31019\_SKOV3\_INF-6HRSUPVSCTRL | Details ... | 277 | -0.43 | -1.23 | 0.000 | 0.336 | 0.711 | 5503 | tags=48%, list=27%, signal=65% |
| 21 | SARRIO\_EPITHELIAL\_MESENCHYMAL\_TRANSITION\_DN |  | 143 | -0.26 | -1.23 | 0.187 | 0.322 | 0.711 | 4443 | tags=28%, list=22%, signal=35% |
| 22 | SANA\_RESPONSE\_TO\_IFNG\_UP |  | 70 | -0.44 | -1.23 | 0.000 | 0.324 | 0.711 | 4581 | tags=43%, list=22%, signal=55% |
| 23 | GROSS\_HIF1A\_TARGETS\_DN |  | 24 | -0.40 | -1.22 | 0.093 | 0.321 | 0.711 | 5165 | tags=50%, list=25%, signal=67% |
| 24 | GSE17708\_A549\_TGFB\_8HRS\_DOWN |  | 219 | -0.40 | -1.22 | 0.000 | 0.309 | 0.711 | 4769 | tags=42%, list=23%, signal=55% |
| 25 | GROSS\_HYPOXIA\_VIA\_ELK3\_DN |  | 152 | -0.44 | -1.22 | 0.091 | 0.320 | 0.711 | 6274 | tags=59%, list=31%, signal=84% |
| 26 | GSE13485\_DAY3\_VS\_DAY7\_YF17D\_VACCINE\_PBMC\_DN |  | 177 | -0.33 | -1.21 | 0.000 | 0.341 | 0.711 | 5331 | tags=38%, list=26%, signal=52% |
| 27 | GSE49329\_LPS UPREGULATED ONLY |  | 218 | -0.27 | -1.21 | 0.184 | 0.360 | 0.711 | 4581 | tags=30%, list=22%, signal=39% |
| 28 | GROSS\_HYPOXIA\_VIA\_HIF1A\_DN |  | 106 | -0.27 | -1.20 | 0.000 | 0.364 | 0.756 | 6616 | tags=50%, list=32%, signal=74% |
| 29 | GSE3920\_IFNA\_VS\_IFNB\_TREATED\_ENDOTHELIAL\_CELL\_UP |  | 162 | -0.22 | -1.20 | 0.000 | 0.353 | 0.756 | 5548 | tags=35%, list=27%, signal=48% |
| 30 | GROSS\_HYPOXIA\_VIA\_ELK3\_UP |  | 201 | -0.38 | -1.19 | 0.214 | 0.352 | 0.756 | 4436 | tags=33%, list=22%, signal=42% |
| 31 | HOSHIDA\_LIVER\_CANCER\_SURVIVAL\_DN |  | 110 | -0.31 | -1.19 | 0.095 | 0.347 | 0.756 | 5567 | tags=43%, list=27%, signal=58% |
| 32 | GSE4748\_CTRL\_VS\_CYANOBACTERIUM\_LPSLIKE\_STIM\_DC\_1H\_UP |  | 193 | -0.36 | -1.19 | 0.000 | 0.342 | 0.756 | 5604 | tags=44%, list=27%, signal=60% |
| 33 | GROSS\_ELK3\_TARGETS\_DN |  | 31 | -0.43 | -1.18 | 0.187 | 0.344 | 0.756 | 3004 | tags=29%, list=15%, signal=34% |
| 34 | GSE3920\_UNTREATED\_VS\_IFNA\_TREATED\_ENDOTHELIAL\_CELL\_UP |  | 167 | -0.41 | -1.18 | 0.000 | 0.337 | 0.756 | 5713 | tags=48%, list=28%, signal=66% |
| 35 | GSE17708\_A549\_TGFB\_2HRS\_DOWN |  | 186 | -0.38 | -1.18 | 0.181 | 0.328 | 0.756 | 4235 | tags=34%, list=21%, signal=42% |
| 36 | GSE3920\_UNTREATED\_VS\_IFNB\_TREATED\_ENDOTHELIAL\_CELL\_DN |  | 165 | -0.49 | -1.18 | 0.091 | 0.323 | 0.756 | 4784 | tags=46%, list=23%, signal=60% |
| 37 | SANA\_RESPONSE\_TO\_IFNG\_DN |  | 82 | -0.60 | -1.17 | 0.127 | 0.357 | 0.756 | 5941 | tags=67%, list=29%, signal=94% |
| 38 | THUM\_SYSTOLIC\_HEART\_FAILURE\_UP |  | 393 | -0.41 | -1.17 | 0.181 | 0.355 | 0.798 | 6061 | tags=49%, list=30%, signal=68% |
| 39 | GSE13485\_CTRL\_VS\_DAY7\_YF17D\_VACCINE\_PBMC\_DN |  | 189 | -0.35 | -1.17 | 0.000 | 0.350 | 0.798 | 5512 | tags=41%, list=27%, signal=55% |
| 40 | GSE4748\_CTRL\_VS\_LPS\_AND\_CYANOBACTERIUM\_LPSLIKE\_STIM\_DC\_3H\_UP |  | 186 | -0.38 | -1.17 | 0.000 | 0.342 | 0.798 | 5698 | tags=46%, list=28%, signal=64% |
| 41 | GSE3920\_UNTREATED\_VS\_IFNA\_TREATED\_ENDOTHELIAL\_CELL\_DN |  | 156 | -0.34 | -1.17 | 0.091 | 0.335 | 0.798 | 4474 | tags=33%, list=22%, signal=42% |
| 42 | GSE31019\_HT1080\_INF-6HRSUPVSCTRL |  | 150 | -0.50 | -1.16 | 0.000 | 0.330 | 0.798 | 6249 | tags=60%, list=31%, signal=86% |
| 43 | GSE3920\_UNTREATED\_VS\_IFNG\_TREATED\_ENDOTHELIAL\_CELL\_DN |  | 171 | -0.43 | -1.16 | 0.218 | 0.331 | 0.798 | 6265 | tags=50%, list=31%, signal=72% |
| 44 | GSE49329\_MURINE MICROGLIA\_IL4\_UP2FOLDS |  | 350 | -0.34 | -1.15 | 0.189 | 0.350 | 0.798 | 5503 | tags=42%, list=27%, signal=56% |
| 45 | GROSS\_HYPOXIA\_VIA\_HIF1A\_UP |  | 74 | -0.55 | -1.15 | 0.091 | 0.350 | 0.798 | 4493 | tags=47%, list=22%, signal=60% |
| 46 | GSE13485\_PRE\_VS\_POST\_YF17D\_VACCINATION\_PBMC\_DN |  | 187 | -0.45 | -1.14 | 0.000 | 0.358 | 0.798 | 5681 | tags=53%, list=28%, signal=73% |
| 47 | GOTZMANN\_EPITHELIAL\_TO\_MESENCHYMAL\_TRANSITION\_DN |  | 202 | -0.37 | -1.14 | 0.127 | 0.356 | 0.798 | 5334 | tags=44%, list=26%, signal=59% |
| 48 | GSE17708\_A549\_TGFB\_0.5HRS\_DOWN |  | 285 | -0.41 | -1.14 | 0.091 | 0.358 | 0.798 | 4644 | tags=42%, list=23%, signal=54% |
| 49 | GROSS\_HYPOXIA\_VIA\_ELK3\_AND\_HIF1A\_DN |  | 100 | -0.40 | -1.14 | 0.091 | 0.354 | 0.798 | 4438 | tags=36%, list=22%, signal=46% |
| 50 | GSE13485\_CTRL\_VS\_DAY1\_YF17D\_VACCINE\_PBMC\_UP |  | 180 | -0.64 | -1.14 | 0.000 | 0.348 | 0.798 | 4501 | tags=65%, list=22%, signal=83% |
| 51 | GSE13485\_DAY3\_VS\_DAY7\_YF17D\_VACCINE\_PBMC\_UP |  | 157 | -0.21 | -1.13 | 0.173 | 0.344 | 0.798 | 6375 | tags=35%, list=31%, signal=51% |
| 52 | GSE3920\_UNTREATED\_VS\_IFNG\_TREATED\_FIBROBLAST\_UP |  | 163 | -0.19 | -1.13 | 0.079 | 0.340 | 0.798 | 4243 | tags=23%, list=21%, signal=29% |
| 53 | JAEGER\_METASTASIS\_UP |  | 41 | -0.38 | -1.13 | 0.335 | 0.335 | 0.798 | 4294 | tags=41%, list=21%, signal=52% |
| 54 | GSE13485\_DAY1\_VS\_DAY7\_YF17D\_VACCINE\_PBMC\_DN |  | 188 | -0.57 | -1.13 | 0.000 | 0.332 | 0.798 | 4856 | tags=56%, list=24%, signal=73% |
| 55 | GSE13485\_CTRL\_VS\_DAY3\_YF17D\_VACCINE\_PBMC\_DN |  | 185 | -0.36 | -1.12 | 0.000 | 0.358 | 0.906 | 5681 | tags=43%, list=28%, signal=59% |
| 56 | CHIANG\_LIVER\_CANCER\_SUBCLASS\_PROLIFERATION\_UP |  | 168 | -0.41 | -1.12 | 0.433 | 0.357 | 0.948 | 4792 | tags=35%, list=23%, signal=45% |
| 57 | GSE3920\_UNTREATED\_VS\_IFNB\_TREATED\_ENDOTHELIAL\_CELL\_UP |  | 179 | -0.30 | -1.12 | 0.189 | 0.351 | 0.948 | 5329 | tags=37%, list=26%, signal=50% |
| 58 | SARRIO\_EPITHELIAL\_MESENCHYMAL\_TRANSITION\_UP |  | 170 | -0.42 | -1.12 | 0.341 | 0.352 | 0.948 | 4986 | tags=40%, list=24%, signal=52% |
| 59 | GSE49329\_PBS-VS-IL4 1.5FOLDS\_FDR005 |  | 120 | -0.25 | -1.11 | 0.377 | 0.371 | 0.948 | 4362 | tags=28%, list=21%, signal=36% |
| 60 | GSE13485\_DAY1\_VS\_DAY3\_YF17D\_VACCINE\_PBMC\_DN |  | 183 | -0.61 | -1.10 | 0.181 | 0.397 | 0.948 | 5125 | tags=67%, list=25%, signal=88% |
| 61 | GSE3920\_IFNB\_VS\_IFNG\_TREATED\_ENDOTHELIAL\_CELL\_DN |  | 172 | -0.45 | -1.09 | 0.091 | 0.410 | 0.948 | 5141 | tags=45%, list=25%, signal=60% |
| 62 | GSE4748\_CTRL\_VS\_CYANOBACTERIUM\_LPSLIKE\_STIM\_DC\_3H\_UP |  | 192 | -0.23 | -1.09 | 0.286 | 0.422 | 0.948 | 5500 | tags=33%, list=27%, signal=45% |
| 63 | LU\_EZH2\_TARGETS\_DN |  | 357 | -0.44 | -1.08 | 0.181 | 0.440 | 1.000 | 5125 | tags=44%, list=25%, signal=57% |
| 64 | CHIANG\_LIVER\_CANCER\_SUBCLASS\_POLYSOMY7\_DN |  | 24 | -0.39 | -1.08 | 0.425 | 0.437 | 1.000 | 4778 | tags=46%, list=23%, signal=60% |
| 65 | GSE49329\_PBS-VS-LPS 5FOLDS\_FDR<0.05 |  | 144 | -0.24 | -1.07 | 0.274 | 0.450 | 1.000 | 4342 | tags=26%, list=21%, signal=33% |
| 66 | VEGF\_A\_UP.V1\_DN |  | 187 | -0.56 | -1.07 | 0.214 | 0.454 | 1.000 | 5115 | tags=57%, list=25%, signal=76% |
| 67 | GSE3920\_UNTREATED\_VS\_IFNA\_TREATED\_FIBROBLAST\_DN |  | 188 | -0.41 | -1.07 | 0.341 | 0.449 | 1.000 | 4489 | tags=36%, list=22%, signal=46% |
| 68 | LU\_EZH2\_TARGETS\_UP |  | 264 | -0.21 | -1.06 | 0.394 | 0.459 | 1.000 | 4727 | tags=25%, list=23%, signal=33% |
| 69 | GSE49329\_MURINE MICROGLIA\_LPS\_DOWN |  | 277 | -0.39 | -1.06 | 0.399 | 0.453 | 1.000 | 4392 | tags=35%, list=21%, signal=44% |
| 70 | GSE4748\_CTRL\_VS\_LPS\_AND\_CYANOBACTERIUM\_LPSLIKE\_STIM\_DC\_3H\_DN |  | 196 | -0.22 | -1.06 | 0.317 | 0.452 | 1.000 | 6478 | tags=40%, list=32%, signal=58% |
| 71 | GSE4748\_CTRL\_VS\_LPS\_STIM\_DC\_3H\_UP |  | 194 | -0.31 | -1.06 | 0.274 | 0.453 | 1.000 | 5724 | tags=40%, list=28%, signal=55% |
| 72 | GSE3920\_UNTREATED\_VS\_IFNA\_TREATED\_FIBROBLAST\_UP |  | 162 | -0.21 | -1.05 | 0.365 | 0.487 | 1.000 | 4361 | tags=25%, list=21%, signal=31% |
| 73 | GROSS\_HYPOXIA\_VIA\_ELK3\_ONLY\_DN |  | 40 | -0.18 | -1.04 | 0.260 | 0.498 | 1.000 | 4436 | tags=23%, list=22%, signal=29% |
| 74 | GSE17708\_A549\_TGFB\_72HRS\_UP |  | 395 | -0.28 | -1.04 | 0.365 | 0.495 | 1.000 | 6599 | tags=45%, list=32%, signal=65% |
| 75 | GSE13485\_CTRL\_VS\_DAY21\_YF17D\_VACCINE\_PBMC\_UP |  | 177 | -0.32 | -1.04 | 0.365 | 0.491 | 1.000 | 6565 | tags=43%, list=32%, signal=63% |
| 76 | GOTZMANN\_EPITHELIAL\_TO\_MESENCHYMAL\_TRANSITION\_UP |  | 66 | -0.31 | -1.04 | 0.381 | 0.501 | 1.000 | 3160 | tags=23%, list=15%, signal=27% |
| 77 | GSE4748\_LPS\_VS\_LPS\_AND\_CYANOBACTERIUM\_LPSLIKE\_STIM\_DC\_3H\_UP |  | 175 | -0.37 | -1.03 | 0.365 | 0.519 | 1.000 | 5513 | tags=42%, list=27%, signal=57% |
| 78 | GSE3920\_IFNA\_VS\_IFNG\_TREATED\_ENDOTHELIAL\_CELL\_UP |  | 156 | -0.34 | -1.03 | 0.365 | 0.531 | 1.000 | 5132 | tags=36%, list=25%, signal=48% |
| 79 | GSE3920\_IFNA\_VS\_IFNG\_TREATED\_FIBROBLAST\_DN |  | 178 | -0.15 | -1.02 | 0.363 | 0.531 | 1.000 | 5747 | tags=28%, list=28%, signal=38% |
| 80 | GSE49329\_MURINE MICROGLIA\_IL4\_UP |  | 254 | -0.28 | -1.01 | 0.365 | 0.559 | 1.000 | 5432 | tags=37%, list=27%, signal=50% |
| 81 | GSE4748\_CTRL\_VS\_CYANOBACTERIUM\_LPSLIKE\_STIM\_DC\_1H\_DN |  | 191 | -0.27 | -1.01 | 0.581 | 0.553 | 1.000 | 4170 | tags=28%, list=20%, signal=35% |
| 82 | BEGUM\_TARGETS\_OF\_PAX3\_FOXO1\_FUSION\_UP |  | 59 | -0.35 | -1.01 | 0.488 | 0.551 | 1.000 | 5362 | tags=37%, list=26%, signal=50% |
| 83 | HOSHIDA\_LIVER\_CANCER\_LATE\_RECURRENCE\_UP |  | 57 | -0.28 | -1.01 | 0.544 | 0.558 | 1.000 | 6282 | tags=40%, list=31%, signal=58% |
| 84 | GSE49329\_IL4 UPREGULATED ONLY |  | 203 | -0.30 | -1.00 | 0.365 | 0.579 | 1.000 | 5531 | tags=41%, list=27%, signal=56% |
| 85 | GSE17708\_A549\_TGFB\_8HRS\_UP |  | 350 | -0.18 | -0.96 | 0.486 | 0.690 | 1.000 | 6457 | tags=35%, list=32%, signal=50% |
| 86 | GSE17708\_A549\_TGFB\_24HRS\_UP |  | 314 | -0.14 | -0.96 | 0.486 | 0.683 | 1.000 | 6240 | tags=32%, list=31%, signal=45% |
| 87 | SANA\_TNF\_SIGNALING\_DN |  | 89 | -0.39 | -0.95 | 0.645 | 0.706 | 1.000 | 5263 | tags=38%, list=26%, signal=51% |
| 88 | GSE3920\_IFNA\_VS\_IFNG\_TREATED\_FIBROBLAST\_UP |  | 168 | -0.18 | -0.92 | 0.456 | 0.782 | 1.000 | 4971 | tags=27%, list=24%, signal=36% |
| 89 | GSE13485\_DAY7\_VS\_DAY21\_YF17D\_VACCINE\_PBMC\_UP |  | 186 | -0.23 | -0.92 | 0.665 | 0.792 | 1.000 | 6249 | tags=36%, list=31%, signal=51% |
| 90 | GSE17708\_A549\_TGFB\_2HRS\_UP |  | 345 | -0.14 | -0.91 | 0.672 | 0.792 | 1.000 | 5269 | tags=24%, list=26%, signal=32% |
| 91 | GSE3920\_IFNA\_VS\_IFNG\_TREATED\_ENDOTHELIAL\_CELL\_DN |  | 161 | -0.15 | -0.89 | 0.737 | 0.821 | 1.000 | 4718 | tags=23%, list=23%, signal=30% |
| 92 | JECHLINGER\_EPITHELIAL\_TO\_MESENCHYMAL\_TRANSITION\_UP |  | 70 | -0.19 | -0.88 | 0.471 | 0.831 | 1.000 | 2188 | tags=13%, list=11%, signal=14% |
| 93 | GSE13485\_DAY3\_VS\_DAY21\_YF17D\_VACCINE\_PBMC\_UP |  | 182 | -0.27 | -0.88 | 0.915 | 0.836 | 1.000 | 4550 | tags=24%, list=22%, signal=30% |
| 94 | CHIANG\_LIVER\_CANCER\_SUBCLASS\_CTNNB1\_UP |  | 168 | -0.18 | -0.87 | 0.484 | 0.830 | 1.000 | 4860 | tags=27%, list=24%, signal=36% |
| 95 | GSE3920\_UNTREATED\_VS\_IFNG\_TREATED\_FIBROBLAST\_DN |  | 171 | -0.20 | -0.86 | 0.825 | 0.835 | 1.000 | 5016 | tags=28%, list=25%, signal=37% |
| 96 | GSE4748\_CYANOBACTERIUM\_LPSLIKE\_VS\_LPS\_AND\_CYANOBACTERIUM\_LPSLIKE\_STIM\_DC\_3H\_UP |  | 173 | -0.16 | -0.84 | 0.672 | 0.863 | 1.000 | 4900 | tags=28%, list=24%, signal=36% |
| 97 | GSE13485\_PRE\_VS\_POST\_YF17D\_VACCINATION\_PBMC\_UP |  | 169 | -0.14 | -0.82 | 0.669 | 0.874 | 1.000 | 4862 | tags=24%, list=24%, signal=31% |
| 98 | THUM\_SYSTOLIC\_HEART\_FAILURE\_DN |  | 211 | -0.26 | -0.82 | 0.915 | 0.876 | 1.000 | 5443 | tags=37%, list=27%, signal=50% |
| 99 | GSE13485\_CTRL\_VS\_DAY7\_YF17D\_VACCINE\_PBMC\_UP |  | 163 | -0.13 | -0.75 | 0.826 | 0.923 | 1.000 | 6099 | tags=33%, list=30%, signal=47% |
| 100 | GO\_POSITIVE\_REGULATION\_OF\_EPITHELIAL\_TO\_MESENCHYMAL\_TRANSITION |  | 33 | -0.20 | -0.75 | 0.920 | 0.916 | 1.000 | 4680 | tags=27%, list=23%, signal=35% |
Table: Gene sets enriched in phenotype **Huvec\_JINB8\_MHC-Exo (3 samples)**[plain text format]****

  
