# Supplementary material for: CD63+ and MHC Class I+ Subsets of Extracellular Vesicles Produced by Wild-Type and CD47-Deficient Jurkat T Cells Have Divergent Functional Effects on Endothelial Cell Gene Expression
Source: Biomedicines. 2021 Nov 17;9(11):1705. doi: 10.3390/biomedicines9111705 (PMC8615535; doi:10.3390/biomedicines9111705)
Supplement: Supplementary file 1 [file biomedicines-09-01705-s001.zip › gsea_report_for_Huvec_JK_CD63-Exo_1539178329650.html]

Report for Huvec\_JK\_CD63-Exo 1539178329650 [GSEA]

| GS  follow link to MSigDB | GS DETAILS | SIZE | ES | NES | NOM p-val | FDR q-val | FWER p-val | RANK AT MAX | LEADING EDGE || 1 | GROSS\_ELK3\_TARGETS\_UP | Details ... | 27 | 0.45 | 1.65 | 0.000 | 0.171 | 0.037 | 6878 | tags=52%, list=34%, signal=78% |
| 2 | SARRIO\_EPITHELIAL\_MESENCHYMAL\_TRANSITION\_UP | Details ... | 170 | 0.56 | 1.58 | 0.000 | 0.204 | 0.239 | 4062 | tags=42%, list=20%, signal=52% |
| 3 | CHIANG\_LIVER\_CANCER\_SUBCLASS\_PROLIFERATION\_UP | Details ... | 168 | 0.57 | 1.56 | 0.000 | 0.151 | 0.239 | 2477 | tags=37%, list=12%, signal=42% |
| 4 | GROSS\_HYPOXIA\_VIA\_ELK3\_AND\_HIF1A\_DN | Details ... | 100 | 0.48 | 1.56 | 0.000 | 0.125 | 0.239 | 5393 | tags=40%, list=26%, signal=54% |
| 5 | GROSS\_HYPOXIA\_VIA\_ELK3\_UP | Details ... | 201 | 0.46 | 1.56 | 0.000 | 0.110 | 0.239 | 6418 | tags=47%, list=31%, signal=67% |
| 6 | SANA\_TNF\_SIGNALING\_DN | Details ... | 89 | 0.46 | 1.52 | 0.000 | 0.158 | 0.382 | 7944 | tags=67%, list=39%, signal=110% |
| 7 | GOTZMANN\_EPITHELIAL\_TO\_MESENCHYMAL\_TRANSITION\_UP | Details ... | 66 | 0.39 | 1.45 | 0.000 | 0.263 | 0.522 | 6663 | tags=50%, list=33%, signal=74% |
| 8 | SANA\_RESPONSE\_TO\_IFNG\_DN | Details ... | 82 | 0.54 | 1.45 | 0.000 | 0.262 | 0.567 | 6730 | tags=65%, list=33%, signal=96% |
| 9 | GSE36476\_CTRL\_VS\_TSST\_ACT\_40H\_MEMORY\_CD4\_TCELL\_YOUNG\_DN | Details ... | 194 | 0.51 | 1.44 | 0.000 | 0.258 | 0.612 | 4674 | tags=49%, list=23%, signal=64% |
| 10 | JAEGER\_METASTASIS\_UP | Details ... | 41 | 0.56 | 1.44 | 0.000 | 0.241 | 0.612 | 4156 | tags=54%, list=20%, signal=67% |
| 11 | GSE14415\_INDUCED\_TREG\_VS\_TCONV\_UP | Details ... | 173 | 0.50 | 1.43 | 0.000 | 0.256 | 0.612 | 2753 | tags=32%, list=13%, signal=37% |
| 12 | VEGF\_A\_UP.V1\_DN | Details ... | 187 | 0.58 | 1.43 | 0.000 | 0.242 | 0.612 | 5695 | tags=66%, list=28%, signal=91% |
| 13 | GROSS\_HYPOXIA\_VIA\_HIF1A\_UP | Details ... | 74 | 0.52 | 1.40 | 0.214 | 0.302 | 0.786 | 6330 | tags=61%, list=31%, signal=88% |
| 14 | GOTZMANN\_EPITHELIAL\_TO\_MESENCHYMAL\_TRANSITION\_DN | Details ... | 202 | 0.33 | 1.39 | 0.000 | 0.291 | 0.786 | 6465 | tags=44%, list=32%, signal=63% |
| 15 | GSE3920\_UNTREATED\_VS\_IFNB\_TREATED\_ENDOTHELIAL\_CELL\_DN | Details ... | 165 | 0.50 | 1.39 | 0.000 | 0.289 | 0.786 | 4812 | tags=48%, list=24%, signal=63% |
| 16 | GSE4748\_CTRL\_VS\_LPS\_AND\_CYANOBACTERIUM\_LPSLIKE\_STIM\_DC\_3H\_DN | Details ... | 196 | 0.28 | 1.39 | 0.000 | 0.284 | 0.786 | 6271 | tags=42%, list=31%, signal=60% |
| 17 | GROSS\_HYPOXIA\_VIA\_ELK3\_AND\_HIF1A\_UP | Details ... | 138 | 0.38 | 1.38 | 0.000 | 0.275 | 0.786 | 6217 | tags=50%, list=30%, signal=71% |
| 18 | GSE4748\_CTRL\_VS\_LPS\_AND\_CYANOBACTERIUM\_LPSLIKE\_STIM\_DC\_3H\_UP | Details ... | 186 | 0.31 | 1.36 | 0.124 | 0.312 | 0.849 | 6336 | tags=45%, list=31%, signal=65% |
| 19 | GSE17708\_A549\_TGFB\_8HRS\_UP | Details ... | 350 | 0.21 | 1.35 | 0.125 | 0.323 | 0.849 | 7493 | tags=43%, list=37%, signal=66% |
| 20 | GSE3920\_UNTREATED\_VS\_IFNA\_TREATED\_FIBROBLAST\_DN | Details ... | 188 | 0.44 | 1.35 | 0.212 | 0.325 | 0.901 | 5503 | tags=44%, list=27%, signal=59% |
| 21 | GSE3920\_UNTREATED\_VS\_IFNG\_TREATED\_ENDOTHELIAL\_CELL\_DN |  | 171 | 0.40 | 1.34 | 0.214 | 0.319 | 0.901 | 6334 | tags=49%, list=31%, signal=70% |
| 22 | GSE4748\_CTRL\_VS\_LPS\_STIM\_DC\_3H\_UP |  | 194 | 0.28 | 1.31 | 0.000 | 0.381 | 0.901 | 6766 | tags=51%, list=33%, signal=76% |
| 23 | GSE17708\_A549\_TGFB\_24HRS\_UP |  | 314 | 0.20 | 1.30 | 0.125 | 0.422 | 0.955 | 8559 | tags=51%, list=42%, signal=86% |
| 24 | GSE4748\_LPS\_VS\_LPS\_AND\_CYANOBACTERIUM\_LPSLIKE\_STIM\_DC\_3H\_UP |  | 175 | 0.33 | 1.30 | 0.212 | 0.413 | 0.955 | 3970 | tags=30%, list=19%, signal=37% |
| 25 | GSE4748\_CTRL\_VS\_CYANOBACTERIUM\_LPSLIKE\_STIM\_DC\_3H\_DN |  | 192 | 0.36 | 1.27 | 0.212 | 0.459 | 1.000 | 6075 | tags=46%, list=30%, signal=65% |
| 26 | CHIANG\_LIVER\_CANCER\_SUBCLASS\_UNANNOTATED\_DN |  | 184 | 0.50 | 1.25 | 0.214 | 0.490 | 1.000 | 7290 | tags=65%, list=36%, signal=100% |
| 27 | GSE3920\_IFNB\_VS\_IFNG\_TREATED\_ENDOTHELIAL\_CELL\_DN |  | 172 | 0.42 | 1.25 | 0.214 | 0.484 | 1.000 | 8032 | tags=67%, list=39%, signal=109% |
| 28 | LU\_EZH2\_TARGETS\_DN |  | 357 | 0.36 | 1.24 | 0.214 | 0.509 | 1.000 | 7520 | tags=59%, list=37%, signal=91% |
| 29 | HU\_ANGIOGENESIS\_DN |  | 37 | 0.41 | 1.21 | 0.000 | 0.567 | 1.000 | 5680 | tags=49%, list=28%, signal=67% |
| 30 | GSE13485\_DAY1\_VS\_DAY3\_YF17D\_VACCINE\_PBMC\_DN |  | 183 | 0.49 | 1.21 | 0.345 | 0.550 | 1.000 | 5946 | tags=54%, list=29%, signal=75% |
| 31 | GSE13485\_DAY1\_VS\_DAY21\_YF17D\_VACCINE\_PBMC\_DN |  | 177 | 0.57 | 1.21 | 0.345 | 0.541 | 1.000 | 4669 | tags=58%, list=23%, signal=75% |
| 32 | GSE13485\_CTRL\_VS\_DAY1\_YF17D\_VACCINE\_PBMC\_UP |  | 180 | 0.54 | 1.20 | 0.345 | 0.576 | 1.000 | 5405 | tags=58%, list=26%, signal=78% |
| 33 | GSE49329\_MURINE MICROGLIA\_LPS\_DOWN |  | 277 | 0.36 | 1.19 | 0.345 | 0.596 | 1.000 | 6490 | tags=49%, list=32%, signal=71% |
| 34 | GSE4748\_CTRL\_VS\_CYANOBACTERIUM\_LPSLIKE\_STIM\_DC\_1H\_UP |  | 193 | 0.28 | 1.19 | 0.286 | 0.586 | 1.000 | 6282 | tags=41%, list=31%, signal=59% |
| 35 | GSE17708\_A549\_TGFB\_0.5HRS\_DOWN |  | 285 | 0.37 | 1.18 | 0.174 | 0.599 | 1.000 | 4348 | tags=39%, list=21%, signal=49% |
| 36 | SANA\_RESPONSE\_TO\_IFNG\_UP |  | 70 | 0.32 | 1.18 | 0.286 | 0.602 | 1.000 | 5159 | tags=34%, list=25%, signal=46% |
| 37 | GSE31019\_HT1080\_INF-6HRSUPVSCTRL |  | 150 | 0.37 | 1.17 | 0.281 | 0.587 | 1.000 | 7265 | tags=51%, list=36%, signal=78% |
| 38 | THUM\_SYSTOLIC\_HEART\_FAILURE\_UP |  | 393 | 0.29 | 1.17 | 0.214 | 0.595 | 1.000 | 6205 | tags=44%, list=30%, signal=62% |
| 39 | GROSS\_HYPOXIA\_VIA\_ELK3\_ONLY\_DN |  | 40 | 0.28 | 1.17 | 0.191 | 0.581 | 1.000 | 3120 | tags=23%, list=15%, signal=26% |
| 40 | GSE13485\_CTRL\_VS\_DAY21\_YF17D\_VACCINE\_PBMC\_UP |  | 177 | 0.28 | 1.16 | 0.286 | 0.579 | 1.000 | 8129 | tags=58%, list=40%, signal=96% |
| 41 | GSE3920\_UNTREATED\_VS\_IFNG\_TREATED\_FIBROBLAST\_DN |  | 171 | 0.24 | 1.16 | 0.182 | 0.597 | 1.000 | 4449 | tags=27%, list=22%, signal=35% |
| 42 | GSE3920\_UNTREATED\_VS\_IFNA\_TREATED\_ENDOTHELIAL\_CELL\_UP |  | 167 | 0.28 | 1.15 | 0.288 | 0.592 | 1.000 | 6129 | tags=39%, list=30%, signal=55% |
| 43 | GROSS\_HYPOXIA\_VIA\_ELK3\_DN |  | 152 | 0.28 | 1.15 | 0.281 | 0.604 | 1.000 | 6159 | tags=38%, list=30%, signal=53% |
| 44 | GSE17708\_A549\_TGFB\_8HRS\_DOWN |  | 219 | 0.34 | 1.15 | 0.178 | 0.593 | 1.000 | 5022 | tags=39%, list=25%, signal=51% |
| 45 | VANOEVELEN\_MYOGENESIS\_SIN3A\_TARGETS |  | 209 | 0.37 | 1.14 | 0.286 | 0.595 | 1.000 | 4026 | tags=29%, list=20%, signal=35% |
| 46 | GSE3920\_IFNA\_VS\_IFNB\_TREATED\_ENDOTHELIAL\_CELL\_UP |  | 162 | 0.26 | 1.14 | 0.091 | 0.593 | 1.000 | 4811 | tags=31%, list=24%, signal=40% |
| 47 | GSE3920\_IFNA\_VS\_IFNG\_TREATED\_ENDOTHELIAL\_CELL\_UP |  | 156 | 0.29 | 1.13 | 0.286 | 0.592 | 1.000 | 5264 | tags=37%, list=26%, signal=49% |
| 48 | GSE13485\_DAY1\_VS\_DAY7\_YF17D\_VACCINE\_PBMC\_DN |  | 188 | 0.42 | 1.13 | 0.417 | 0.601 | 1.000 | 2873 | tags=28%, list=14%, signal=32% |
| 49 | GSE4748\_CTRL\_VS\_CYANOBACTERIUM\_LPSLIKE\_STIM\_DC\_1H\_DN |  | 191 | 0.23 | 1.12 | 0.174 | 0.602 | 1.000 | 6403 | tags=42%, list=31%, signal=61% |
| 50 | THUM\_SYSTOLIC\_HEART\_FAILURE\_DN |  | 211 | 0.32 | 1.12 | 0.313 | 0.593 | 1.000 | 6103 | tags=44%, list=30%, signal=62% |
| 51 | GSE13485\_PRE\_VS\_POST\_YF17D\_VACCINATION\_PBMC\_DN |  | 187 | 0.34 | 1.12 | 0.417 | 0.593 | 1.000 | 7174 | tags=53%, list=35%, signal=82% |
| 52 | GSE13485\_CTRL\_VS\_DAY7\_YF17D\_VACCINE\_PBMC\_DN |  | 189 | 0.27 | 1.11 | 0.286 | 0.605 | 1.000 | 7174 | tags=47%, list=35%, signal=72% |
| 53 | GROSS\_ELK3\_TARGETS\_DN |  | 31 | 0.31 | 1.10 | 0.297 | 0.615 | 1.000 | 3291 | tags=29%, list=16%, signal=35% |
| 54 | GSE13485\_CTRL\_VS\_DAY3\_YF17D\_VACCINE\_PBMC\_DN |  | 185 | 0.26 | 1.09 | 0.283 | 0.636 | 1.000 | 7174 | tags=44%, list=35%, signal=68% |
| 55 | GSE17708\_A549\_TGFB\_24HRS\_DOWN |  | 303 | 0.28 | 1.09 | 0.407 | 0.647 | 1.000 | 5045 | tags=36%, list=25%, signal=47% |
| 56 | GSE3920\_UNTREATED\_VS\_IFNG\_TREATED\_FIBROBLAST\_UP |  | 163 | 0.21 | 1.09 | 0.211 | 0.636 | 1.000 | 5739 | tags=33%, list=28%, signal=45% |
| 57 | GSE17708\_A549\_TGFB\_2HRS\_UP |  | 345 | 0.16 | 1.08 | 0.199 | 0.627 | 1.000 | 5780 | tags=28%, list=28%, signal=38% |
| 58 | CHIANG\_LIVER\_CANCER\_SUBCLASS\_POLYSOMY7\_DN |  | 24 | 0.36 | 1.08 | 0.529 | 0.623 | 1.000 | 5441 | tags=54%, list=27%, signal=74% |
| 59 | GSE49329\_IL4 UPREGULATED ONLY |  | 203 | 0.32 | 1.08 | 0.427 | 0.619 | 1.000 | 4802 | tags=39%, list=23%, signal=50% |
| 60 | GSE22886\_CTRL\_VS\_LPS\_24H\_DC\_DN |  | 188 | 0.32 | 1.08 | 0.282 | 0.617 | 1.000 | 7421 | tags=53%, list=36%, signal=82% |
| 61 | GSE17708\_A549\_TGFB\_72HRS\_UP |  | 395 | 0.23 | 1.07 | 0.354 | 0.622 | 1.000 | 6306 | tags=38%, list=31%, signal=53% |
| 62 | GSE49329\_MURINE MICROGLIA\_IL4\_UP |  | 254 | 0.27 | 1.06 | 0.417 | 0.629 | 1.000 | 4768 | tags=33%, list=23%, signal=42% |
| 63 | GSE49329\_PBS-VS-IL4 1.5FOLDS\_FDR005 |  | 120 | 0.25 | 1.06 | 0.488 | 0.630 | 1.000 | 4768 | tags=33%, list=23%, signal=42% |
| 64 | GSE3920\_UNTREATED\_VS\_IFNA\_TREATED\_ENDOTHELIAL\_CELL\_DN |  | 156 | 0.27 | 1.06 | 0.345 | 0.627 | 1.000 | 3522 | tags=25%, list=17%, signal=30% |
| 65 | GSE4748\_CTRL\_VS\_CYANOBACTERIUM\_LPSLIKE\_STIM\_DC\_3H\_UP |  | 192 | 0.22 | 1.06 | 0.376 | 0.621 | 1.000 | 6729 | tags=37%, list=33%, signal=55% |
| 66 | GSE49329\_MURINE MICROGLIA\_IL4\_UP2FOLDS |  | 350 | 0.27 | 1.04 | 0.417 | 0.645 | 1.000 | 6282 | tags=37%, list=31%, signal=53% |
| 67 | GSE31019\_SKOV3\_INF-6HRSUDOWNVSCTRL |  | 88 | 0.32 | 1.04 | 0.193 | 0.648 | 1.000 | 2797 | tags=28%, list=14%, signal=33% |
| 68 | GSE3920\_IFNA\_VS\_IFNG\_TREATED\_ENDOTHELIAL\_CELL\_DN |  | 161 | 0.20 | 1.03 | 0.268 | 0.655 | 1.000 | 4063 | tags=22%, list=20%, signal=27% |
| 69 | GSE13485\_DAY1\_VS\_DAY21\_YF17D\_VACCINE\_PBMC\_UP |  | 167 | 0.19 | 1.01 | 0.490 | 0.674 | 1.000 | 8202 | tags=50%, list=40%, signal=83% |
| 70 | GROSS\_HYPOXIA\_VIA\_HIF1A\_DN |  | 106 | 0.21 | 1.00 | 0.403 | 0.670 | 1.000 | 4429 | tags=27%, list=22%, signal=35% |
| 71 | GSE17708\_A549\_TGFB\_72HRS\_DOWN |  | 299 | 0.22 | 1.00 | 0.400 | 0.679 | 1.000 | 5078 | tags=32%, list=25%, signal=42% |
| 72 | GSE13485\_PRE\_VS\_POST\_YF17D\_VACCINATION\_PBMC\_UP |  | 169 | 0.20 | 0.99 | 0.410 | 0.676 | 1.000 | 5107 | tags=30%, list=25%, signal=40% |
| 73 | GSE3920\_UNTREATED\_VS\_IFNA\_TREATED\_FIBROBLAST\_UP |  | 162 | 0.23 | 0.99 | 0.520 | 0.670 | 1.000 | 7187 | tags=43%, list=35%, signal=66% |
| 74 | GSE3920\_UNTREATED\_VS\_IFNB\_TREATED\_ENDOTHELIAL\_CELL\_UP |  | 179 | 0.22 | 0.99 | 0.450 | 0.665 | 1.000 | 4239 | tags=27%, list=21%, signal=34% |
| 75 | GSE3920\_IFNA\_VS\_IFNG\_TREATED\_FIBROBLAST\_UP |  | 168 | 0.20 | 0.99 | 0.417 | 0.660 | 1.000 | 2115 | tags=14%, list=10%, signal=15% |
| 76 | GSE3920\_IFNB\_VS\_IFNG\_TREATED\_ENDOTHELIAL\_CELL\_UP |  | 158 | 0.22 | 0.98 | 0.386 | 0.657 | 1.000 | 4420 | tags=25%, list=22%, signal=32% |
| 77 | GSE13485\_DAY3\_VS\_DAY21\_YF17D\_VACCINE\_PBMC\_UP |  | 182 | 0.23 | 0.97 | 0.521 | 0.664 | 1.000 | 6582 | tags=39%, list=32%, signal=57% |
| 78 | GSE4748\_CYANOBACTERIUM\_LPSLIKE\_VS\_LPS\_AND\_CYANOBACTERIUM\_LPSLIKE\_STIM\_DC\_3H\_UP |  | 173 | 0.20 | 0.95 | 0.580 | 0.693 | 1.000 | 5156 | tags=34%, list=25%, signal=44% |
| 79 | GSE17708\_A549\_TGFB\_2HRS\_DOWN |  | 186 | 0.25 | 0.95 | 0.622 | 0.697 | 1.000 | 4070 | tags=31%, list=20%, signal=38% |
| 80 | GSE13485\_DAY7\_VS\_DAY21\_YF17D\_VACCINE\_PBMC\_UP |  | 186 | 0.18 | 0.94 | 0.604 | 0.690 | 1.000 | 8533 | tags=49%, list=42%, signal=84% |
| 81 | GSE49329\_MURINE MICROGLIA\_IL4\_DOWN |  | 127 | 0.21 | 0.93 | 0.514 | 0.712 | 1.000 | 5743 | tags=31%, list=28%, signal=44% |
| 82 | GSE31019\_SKOV3\_INF-6HRSUPVSCTRL |  | 277 | 0.25 | 0.91 | 0.605 | 0.726 | 1.000 | 7129 | tags=43%, list=35%, signal=65% |
| 83 | GSE31019\_HT1080\_INF-12HRSUPVSCTRL |  | 76 | 0.35 | 0.91 | 0.605 | 0.719 | 1.000 | 8470 | tags=64%, list=41%, signal=110% |
| 84 | GSE13485\_DAY3\_VS\_DAY7\_YF17D\_VACCINE\_PBMC\_DN |  | 177 | 0.23 | 0.90 | 0.498 | 0.732 | 1.000 | 6896 | tags=42%, list=34%, signal=63% |
| 85 | MTOR\_UP.N4.V1\_UP |  | 191 | 0.27 | 0.87 | 0.623 | 0.770 | 1.000 | 5068 | tags=34%, list=25%, signal=44% |
| 86 | GSE3920\_IFNA\_VS\_IFNG\_TREATED\_FIBROBLAST\_DN |  | 178 | 0.17 | 0.86 | 0.684 | 0.787 | 1.000 | 2925 | tags=16%, list=14%, signal=19% |
| 87 | CHIANG\_LIVER\_CANCER\_SUBCLASS\_UNANNOTATED\_UP |  | 75 | 0.19 | 0.84 | 0.699 | 0.797 | 1.000 | 2406 | tags=16%, list=12%, signal=18% |
| 88 | GROSS\_HYPOXIA\_VIA\_ELK3\_ONLY\_UP |  | 33 | 0.22 | 0.83 | 0.807 | 0.817 | 1.000 | 4023 | tags=18%, list=20%, signal=23% |
| 89 | SANA\_TNF\_SIGNALING\_UP |  | 78 | 0.27 | 0.80 | 0.604 | 0.840 | 1.000 | 5537 | tags=31%, list=27%, signal=42% |
| 90 | GO\_POSITIVE\_REGULATION\_OF\_EPITHELIAL\_TO\_MESENCHYMAL\_TRANSITION |  | 33 | 0.21 | 0.79 | 0.828 | 0.848 | 1.000 | 1249 | tags=12%, list=6%, signal=13% |
| 91 | JECHLINGER\_EPITHELIAL\_TO\_MESENCHYMAL\_TRANSITION\_DN |  | 66 | 0.17 | 0.77 | 0.801 | 0.862 | 1.000 | 3305 | tags=20%, list=16%, signal=23% |
| 92 | SARRIO\_EPITHELIAL\_MESENCHYMAL\_TRANSITION\_DN |  | 143 | 0.15 | 0.76 | 0.810 | 0.855 | 1.000 | 3690 | tags=17%, list=18%, signal=20% |
| 93 | BEGUM\_TARGETS\_OF\_PAX3\_FOXO1\_FUSION\_UP |  | 59 | 0.16 | 0.76 | 0.687 | 0.853 | 1.000 | 4057 | tags=19%, list=20%, signal=23% |
| 94 | GSE49329\_LPS UPREGULATED ONLY |  | 218 | 0.17 | 0.74 | 0.908 | 0.859 | 1.000 | 1665 | tags=11%, list=8%, signal=12% |
| 95 | GSE49329\_PBS-VS-LPS 5FOLDS\_FDR<0.05 |  | 144 | 0.19 | 0.71 | 0.908 | 0.876 | 1.000 | 1693 | tags=13%, list=8%, signal=14% |
| 96 | JECHLINGER\_EPITHELIAL\_TO\_MESENCHYMAL\_TRANSITION\_UP |  | 70 | 0.14 | 0.68 | 0.908 | 0.895 | 1.000 | 1661 | tags=9%, list=8%, signal=9% |
| 97 | LU\_EZH2\_TARGETS\_UP |  | 264 | 0.13 | 0.65 | 0.906 | 0.909 | 1.000 | 6254 | tags=30%, list=31%, signal=42% |
Table: Gene sets enriched in phenotype **Huvec\_JK\_CD63-Exo (3 samples)**[plain text format]****

  
