# Supplementary material for: CD63+ and MHC Class I+ Subsets of Extracellular Vesicles Produced by Wild-Type and CD47-Deficient Jurkat T Cells Have Divergent Functional Effects on Endothelial Cell Gene Expression
Source: Biomedicines. 2021 Nov 17;9(11):1705. doi: 10.3390/biomedicines9111705 (PMC8615535; doi:10.3390/biomedicines9111705)
Supplement: Supplementary file 1 [file biomedicines-09-01705-s001.zip › gsea_report_for_Huvec_JK_MHC-Exo_1539178433246.html]

Report for Huvec\_JK\_MHC-Exo 1539178433246 [GSEA]

| GS  follow link to MSigDB | GS DETAILS | SIZE | ES | NES | NOM p-val | FDR q-val | FWER p-val | RANK AT MAX | LEADING EDGE || 1 | CHIANG\_LIVER\_CANCER\_SUBCLASS\_POLYSOMY7\_UP | Details ... | 74 | 0.39 | 1.14 | 0.000 | 1.000 | 0.744 | 5405 | tags=41%, list=26%, signal=55% |
| 2 | PIGF\_UP.V1\_DN | Details ... | 182 | 0.46 | 1.09 | 0.000 | 1.000 | 0.937 | 7248 | tags=62%, list=35%, signal=94% |
| 3 | THUM\_MIR21\_TARGETS\_HEART\_DISEASE\_UP | Details ... | 16 | 0.43 | 1.06 | 0.089 | 1.000 | 1.000 | 7851 | tags=63%, list=38%, signal=101% |
| 4 | GSE4748\_LPS\_VS\_LPS\_AND\_CYANOBACTERIUM\_LPSLIKE\_STIM\_DC\_3H\_DN | Details ... | 150 | 0.36 | 1.05 | 0.179 | 1.000 | 1.000 | 6669 | tags=45%, list=33%, signal=66% |
| 5 | GSE13485\_DAY1\_VS\_DAY3\_YF17D\_VACCINE\_PBMC\_UP | Details ... | 158 | 0.49 | 1.05 | 0.250 | 1.000 | 1.000 | 6828 | tags=62%, list=33%, signal=92% |
| 6 | GSE13485\_CTRL\_VS\_DAY1\_YF17D\_VACCINE\_PBMC\_DN | Details ... | 143 | 0.51 | 1.02 | 0.397 | 1.000 | 1.000 | 7153 | tags=66%, list=35%, signal=100% |
| 7 | GSE13485\_DAY1\_VS\_DAY7\_YF17D\_VACCINE\_PBMC\_UP | Details ... | 158 | 0.48 | 1.02 | 0.250 | 1.000 | 1.000 | 7270 | tags=63%, list=36%, signal=96% |
| 8 | GSE17708\_A549\_TGFB\_0.5HRS\_UP | Details ... | 373 | 0.21 | 1.01 | 0.393 | 1.000 | 1.000 | 6226 | tags=35%, list=30%, signal=49% |
| 9 | HOSHIDA\_LIVER\_CANCER\_LATE\_RECURRENCE\_DN | Details ... | 68 | 0.23 | 1.00 | 0.361 | 1.000 | 1.000 | 3697 | tags=26%, list=18%, signal=32% |
| 10 | GO\_EPITHELIAL\_TO\_MESENCHYMAL\_TRANSITION | Details ... | 56 | 0.32 | 1.00 | 0.484 | 1.000 | 1.000 | 7613 | tags=50%, list=37%, signal=79% |
| 11 | GO\_MESENCHYMAL\_TO\_EPITHELIAL\_TRANSITION | Details ... | 15 | 0.61 | 0.99 | 0.698 | 1.000 | 1.000 | 6108 | tags=73%, list=30%, signal=104% |
| 12 | JAEGER\_METASTASIS\_DN | Details ... | 253 | 0.39 | 0.98 | 0.704 | 1.000 | 1.000 | 6409 | tags=45%, list=31%, signal=64% |
| 13 | GSE3920\_IFNA\_VS\_IFNB\_TREATED\_ENDOTHELIAL\_CELL\_DN | Details ... | 157 | 0.18 | 0.95 | 0.652 | 1.000 | 1.000 | 4641 | tags=22%, list=23%, signal=28% |
| 14 | AIGNER\_ZEB1\_TARGETS | Details ... | 33 | 0.33 | 0.94 | 0.825 | 1.000 | 1.000 | 7822 | tags=55%, list=38%, signal=88% |
| 15 | GSE13485\_DAY7\_VS\_DAY21\_YF17D\_VACCINE\_PBMC\_DN | Details ... | 152 | 0.43 | 0.94 | 0.915 | 1.000 | 1.000 | 4778 | tags=46%, list=23%, signal=60% |
| 16 | GO\_CARDIAC\_EPITHELIAL\_TO\_MESENCHYMAL\_TRANSITION | Details ... | 24 | 0.28 | 0.93 | 0.702 | 1.000 | 1.000 | 6562 | tags=42%, list=32%, signal=61% |
| 17 | GSE13485\_DAY3\_VS\_DAY21\_YF17D\_VACCINE\_PBMC\_DN | Details ... | 150 | 0.40 | 0.93 | 0.915 | 1.000 | 1.000 | 4861 | tags=45%, list=24%, signal=59% |
| 18 | CHIANG\_LIVER\_CANCER\_SUBCLASS\_UNANNOTATED\_UP | Details ... | 75 | 0.22 | 0.92 | 0.751 | 1.000 | 1.000 | 2961 | tags=20%, list=14%, signal=23% |
| 19 | CHIANG\_LIVER\_CANCER\_SUBCLASS\_INTERFERON\_DN | Details ... | 45 | 0.30 | 0.92 | 0.609 | 1.000 | 1.000 | 5558 | tags=47%, list=27%, signal=64% |
| 20 | CHIANG\_LIVER\_CANCER\_SUBCLASS\_PROLIFERATION\_DN | Details ... | 173 | 0.30 | 0.91 | 0.607 | 1.000 | 1.000 | 4924 | tags=32%, list=24%, signal=42% |
| 21 | GO\_NEGATIVE\_REGULATION\_OF\_EPITHELIAL\_TO\_MESENCHYMAL\_TRANSITION |  | 22 | 0.37 | 0.88 | 0.575 | 1.000 | 1.000 | 6556 | tags=55%, list=32%, signal=80% |
| 22 | GSE4748\_CYANOBACTERIUM\_LPSLIKE\_VS\_LPS\_AND\_CYANOBACTERIUM\_LPSLIKE\_STIM\_DC\_3H\_DN |  | 168 | 0.26 | 0.87 | 0.734 | 1.000 | 1.000 | 6971 | tags=41%, list=34%, signal=62% |
| 23 | GSE3920\_UNTREATED\_VS\_IFNG\_TREATED\_ENDOTHELIAL\_CELL\_UP |  | 147 | 0.23 | 0.85 | 0.825 | 1.000 | 1.000 | 5337 | tags=31%, list=26%, signal=42% |
| 24 | HOSHIDA\_LIVER\_CANCER\_SURVIVAL\_UP |  | 73 | 0.11 | 0.84 | 0.714 | 1.000 | 1.000 | 8993 | tags=45%, list=44%, signal=80% |
| 25 | GSE13485\_CTRL\_VS\_DAY21\_YF17D\_VACCINE\_PBMC\_DN |  | 147 | 0.31 | 0.84 | 0.915 | 1.000 | 1.000 | 6198 | tags=50%, list=30%, signal=72% |
| 26 | VEGF\_A\_UP.V1\_UP |  | 188 | 0.26 | 0.83 | 0.915 | 1.000 | 1.000 | 7706 | tags=42%, list=38%, signal=67% |
| 27 | CHIANG\_LIVER\_CANCER\_SUBCLASS\_INTERFERON\_UP |  | 26 | 0.20 | 0.77 | 0.847 | 1.000 | 1.000 | 3658 | tags=23%, list=18%, signal=28% |
| 28 | BEGUM\_TARGETS\_OF\_PAX3\_FOXO1\_FUSION\_DN |  | 45 | 0.31 | 0.76 | 0.825 | 1.000 | 1.000 | 8850 | tags=56%, list=43%, signal=98% |
| 29 | GO\_REGULATION\_OF\_EPITHELIAL\_TO\_MESENCHYMAL\_TRANSITION |  | 65 | 0.14 | 0.75 | 0.685 | 1.000 | 1.000 | 7674 | tags=42%, list=38%, signal=66% |
| 30 | GSE13485\_DAY1\_VS\_DAY21\_YF17D\_VACCINE\_PBMC\_UP |  | 167 | 0.11 | 0.74 | 0.831 | 1.000 | 1.000 | 7744 | tags=31%, list=38%, signal=50% |
| 31 | GSE13485\_CTRL\_VS\_DAY3\_YF17D\_VACCINE\_PBMC\_UP |  | 153 | 0.18 | 0.73 | 0.915 | 1.000 | 1.000 | 5337 | tags=31%, list=26%, signal=41% |
| 32 | CHIANG\_LIVER\_CANCER\_SUBCLASS\_CTNNB1\_DN |  | 163 | 0.18 | 0.72 | 0.915 | 1.000 | 1.000 | 6497 | tags=36%, list=32%, signal=52% |
| 33 | JECHLINGER\_EPITHELIAL\_TO\_MESENCHYMAL\_TRANSITION\_DN |  | 66 | 0.17 | 0.62 | 0.915 | 1.000 | 1.000 | 7623 | tags=38%, list=37%, signal=60% |
| 34 | GROSS\_ELK3\_TARGETS\_UP |  | 27 | 0.12 | 0.61 | 0.911 | 0.983 | 1.000 | 2088 | tags=7%, list=10%, signal=8% |
Table: Gene sets enriched in phenotype **Huvec\_JK\_MHC-Exo (3 samples)**[plain text format]****

  
